# The Fall Armyworm *Spodoptera frugiperda* Utilizes Specific UDP-Glycosyltransferases to Inactivate Maize Defensive Benzoxazinoids

**DOI:** 10.3389/fphys.2020.604754

**Published:** 2020-12-21

**Authors:** Bhawana Israni, Felipe C. Wouters, Katrin Luck, Elena Seibel, Seung-Joon Ahn, Christian Paetz, Maximilian Reinert, Heiko Vogel, Matthias Erb, David G. Heckel, Jonathan Gershenzon, Daniel Giddings Vassão

**Affiliations:** ^1^Max Planck Institute for Chemical Ecology, Jena, Germany; ^2^Department of Chemistry, Federal University of São Carlos, São Carlos, Brazil; ^3^Department of Biochemistry, Molecular Biology, Entomology and Plant Pathology, Mississippi State University, Mississippi State, MS, United States; ^4^Institute of Plant Sciences, University of Bern, Bern, Switzerland

**Keywords:** benzoxazinoid, DIMBOA, glucosylation, UGT, *Spodoptera frugiperda* (fall armyworm), detoxification

## Abstract

The relationship between plants and insects is continuously evolving, and many insects rely on biochemical strategies to mitigate the effects of toxic chemicals in their food plants, allowing them to feed on well-defended plants. *Spodoptera frugiperda*, the fall armyworm (FAW), accepts a number of plants as hosts, and has particular success on plants of the Poaceae family such as maize, despite their benzoxazinoid (BXD) defenses. BXDs stored as inert glucosides are converted into toxic aglucones by plant glucosidases upon herbivory. DIMBOA, the main BXD aglucone released by maize leaves, can be stereoselectively re-glucosylated by UDP-glycosyltransferases (UGTs) in the insect gut, rendering it non-toxic. Here, we identify UGTs involved in BXD detoxification by FAW larvae and examine how RNAi-mediated manipulation of the larval glucosylation capacity toward the major maize BXD, DIMBOA, affects larval growth. Our findings highlight the involvement of members of two major UGT families, UGT33 and UGT40, in the glycosylation of BXDs. Most of the BXD excretion in the frass occurs in the form of glucosylated products. Furthermore, the DIMBOA-associated activity was enriched in the gut tissue, with a single conserved UGT33 enzyme (SfUGT33F28) being dedicated to DIMBOA re-glucosylation in the FAW gut. The knock-down of its encoding gene reduces larval performance in a strain-specific manner. This study thus reveals that a single UGT enzyme is responsible for detoxification of the major maize-defensive BXD in this pest insect.

## Introduction

Herbivores must cope with varied plant chemical defenses in order to use plant tissues as a food source. To this end, many herbivorous insects have developed strategies such as behavioral avoidance, rapid excretion, target insensitivity, and biochemical detoxification (Despres et al., 2007). The most common metabolic pathways used to detoxify xenobiotics include functional group activation by enzymes such as cytochrome P450 monooxygenases and esterases (Phase I metabolism), and conjugation with polar residues such as glutathione and sugars (Phase II metabolism) (Kennedy and Tierney, 2013). UDP-glycosyltransferase (UGT) enzymes using uridine diphosphate glucose (UDP-Glc) as a sugar donor play a crucial role for sugar conjugation in insects (Ahn et al., 2012). Conjugation to glucose increases the hydrophilicity of metabolites, restricting their diffusion across membranes and facilitating their recognition and excretion by transport systems (Phase III metabolism). Glycosylation of plant defensive metabolites therefore represents an effective way to control their stability, subcellular localization, bioavailability, and activity (Meech et al., 2012), and constitutes an important route for their detoxification by insects.

The increasing number of available genome and transcriptome resources has allowed the investigation of insect UGTs on a molecular level. The identification of all putative UGTs from *B. mori* and *H. armigera*, together with a comparative analysis of 310 UGTs from genome databases, has provided unprecedented insights on the phylogeny of insect UGTs (Huang et al., 2008; Ahn et al., 2012). Similar to human UGTs, insect UGTs are membrane-bound and present a relatively conserved *C*-terminal domain responsible for binding to the sugar donor, and a more variable *N*-terminal domain, which contains the catalytic residues and is responsible for substrate specificity (Ahn et al., 2012; Krempl et al., 2016). Nevertheless, characterization of insect UGTs, especially at the functional level, is still scarce in the literature, and this superfamily of detoxifying enzymes is not as well studied as others such as cytochrome P450 monooxygenases, glutathione-*S*-transferases (GSTs), and carboxylesterases (Despres et al., 2007). Some insect UGTs, for example, are known to participate in cuticle formation (Kramer and Hopkins, 1987; Hopkins and Kramer, 1992), pigmentation (Daimon et al., 2010), and olfaction and odorant modulation (Wang et al., 1999; Wang et al., 2018). Insect viruses possess genes encoding ecdysteroid glucosyltransferases that are assumed to be derived from insect UGT genes, and are involved with larval molting delay during infection (O’Reilly and Miller, 1989) and behavior modulation (Hoover et al., 2011). In addition, insect UGTs are expected to play important roles in the metabolism of xenobiotics, including plant defenses (Heidel-Fischer and Vogel, 2015) and insecticides (Bull and Whitten, 1972; Li et al., 2018). In *Manduca sexta*, a phenol glucosyltransferase was found to confer tolerance to ingested plant phenolics (Ahmad and Hopkins, 1993), while a glucosyltransferase from the silkworm *Bombyx mori* was found to be active toward a wide range of substrates, including flavonoids and phenols (Luque and O’Reilly, 2002). Several heliothine moths metabolize capsaicin via glucosylation (Ahn et al., 2011), and some of these species also use particular UGTs to aid in detoxification of the cotton toxin gossypol (Krempl et al., 2016).

Many grasses (Poaceae), including crops such as wheat, rye, and maize, accumulate benzoxazinoids (BXDs). The BXD family comprises approximately 20 compounds that share a 2-hydroxy-2*H*-1,4-benzoxazin-3(4*H*)-one core (Frey et al., 1997, 2009). They make up a crucial component of the defense repertoire of these plants against herbivores (Sicker and Schulz, 2002; Niemeyer, 2009), while also functioning in the recruitment of beneficial rhizobacteria (Neal et al., 2012), modulation of auxin signaling (Hasegawa et al., 1992; Nakajima et al., 2001), and iron acquisition (Hu et al., 2018), to name a few. These indole-derived compounds are stored as inert glucosides, and upon tissue damage by a chewing herbivore they are hydrolyzed by specific plant β-glucosidases releasing toxic aglucones (Wouters et al., 2016a,b; Zhou et al., 2018). Some *Spodoptera* larvae re-glucosylate the toxic aglucone DIMBOA derived from the most abundant BXD glucoside in maize leaves, (2*R*)-DIMBOA-Glc, as a detoxification strategy (Wouters et al., 2014). The stereoselectivity of the insect-catalyzed conjugation renders the new glucoside, (*2S*)-DIMBOA-Glc, inert toward the BXD-activating plant β-glucosidases, which survive the larval gut environment but can only hydrolyze the plant-derived (*2R*)-DIMBOA-Glc (Glauser et al., 2011; Wouters et al., 2014; Vassão et al., 2018). In essence, both insect- and plant-derived UGTs use BXDs and UDP-Glc as substrates, but perform their glucosylation reactions to produce a different final stereochemistry. Glucosylation of BXDs had been previously observed in lepidopterans such as *Spodoptera frugiperda*, *Spodoptera littoralis*, *Mythimna separata*, and *Ostrinia furnacalis* (Sasai et al., 2009; Kojima et al., 2010; Glauser et al., 2011; Phuong et al., 2016), but the enzymes responsible remain to be identified. Furthermore, *N*-glucosylation of MBOA, a toxic spontaneous degradation product of many BXDs, has been described as a detoxification mechanism in *S. frugiperda* and *S. littoralis* (Maag et al., 2014) as well as in the coleopteran *Diabrotica virgifera virgifera* (Robert et al., 2017). The latter also uses MBOA-Glc as a deterrent against its own natural enemies (Robert et al., 2017; Zhang et al., 2019).

The fall armyworm (FAW) *S. frugiperda* is a serious pest of maize that has invaded and subsequently spread through Sub-Saharan Africa since 2016 causing enormous crop losses (Goergen et al., 2016; Day et al., 2017), and has more recently entered South Asia, where it is also dispersing rapidly (Swamy et al., 2018). Two ecological strains of FAW have been recognized from natural populations, the so-called corn and rice strains (Pashley, 1986; Nagoshi et al., 2007). Corn strain insects are prevalent on grasses such as maize and sorghum, while the rice strain appears to predominate on small grasses such as rice and Bermuda grass. In most fields, both strains seem to co-occur in varying proportions, and while they are morphologically indistinguishable, they exhibit differential host plant usage and show some reproductive isolation (Groot et al., 2010; Dumas et al., 2015), as well as constitutive transcriptional differences, particularly in relation to mitochondrial transcription (Lu and Adang, 1996; Juárez et al., 2012; Orsucci et al., 2020). The recent sequencing of this species’ genome has opened new possibilities to understand UGT functions *in vivo*, and how these enzymes might be involved in adaptive evolution in this pest insect (Gouin et al., 2017). However, the enzymes used by this insect for BXD glucosylation have not been identified.

In the present work, we analyze the fate of ingested BXDs in *S. frugiperda*, confirming that glucosylation plays a quantitatively major role in BXD metabolism in this insect. We also explore the activities of UGTs produced in different *S. frugiperda* strains and tissues toward DIMBOA and MBOA and identify enzymes with substrate-specific UGT activities. Comparisons of *in vivo* gene expression and UGT activities in specific tissues, together with screening of activity of recombinant enzymes *in vitro*, reveals a DIMBOA-specific role for a single *S. frugiperda* UGT. Its closest homolog in the related lepidopteran *S. littoralis* is shown to be active toward DIMBOA, suggesting the likely conservation of the glucosylation pathway in this genus. Silencing the expression of the gene encoding this particular UGT leads to compromised BXD glycosylation in cultured cells and whole insects, and reduces larval development. These combined results highlight the importance of this UGT to FAW, as it allows this pest to overcome the major chemical defense metabolites of maize through stereoselective glycosylation.

## Results

### Glucosylation Is the Major Pathway of BXD Metabolism in FAW

**FIGURE 1:**
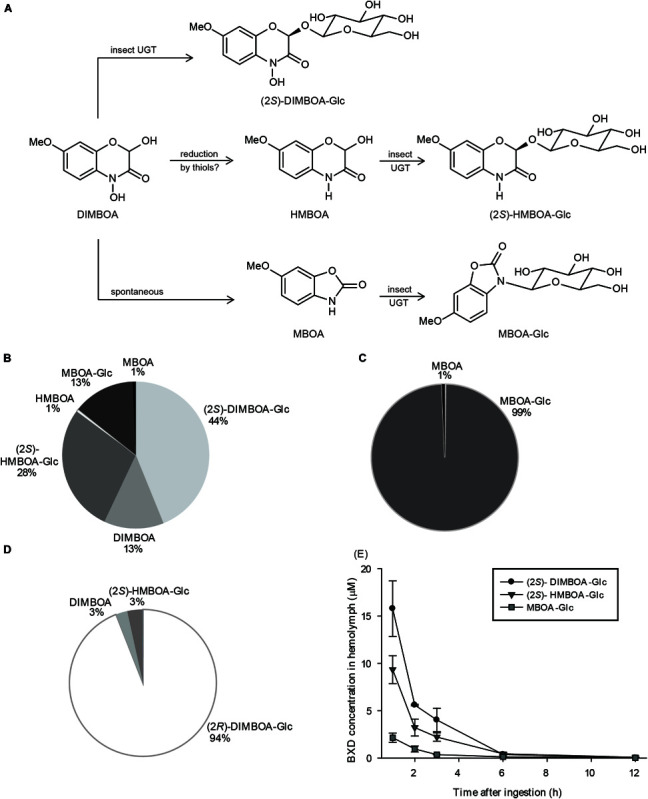
Fate of DIMBOA and associated benzoxazinoid metabolites in *S. frugiperda*. **(A)** Schematic representation of DIMBOA metabolism in *S. frugiperda*. Quantification of BXD metabolites recovered in *S. frugiperda* frass after oral administration of **(B)** DIMBOA, **(C)** MBOA, **(D)** (2*R*)-DIMBOA-Glc (*n* = 5–6, mean ± SEM), and **(E)** BXD glucosides detected by HPLC-MS/MS in *S. frugiperda* hemolymph after oral administration of DIMBOA (*n* = 3, mean ± SEM).

The metabolism of isolated BXDs in FAW was quantified by feeding droplets of their solutions in sugary water to fourth/fifth instar larvae, and analyzing their frass via HPLC-MS/MS after additional feeding on artificial diet without BXDs for 24 h. Through this method, 30 – 55% of the administered BXDs could be recovered in the frass (Supplementary Figures S1A–C). The overall fate of DIMBOA is illustrated in Figure 1A. Among the compounds recovered, the most abundant insect-derived product, (2*S*)-DIMBOA-Glc (44% of the recovered BXD pool) originated from glucosylation of DIMBOA, while some DIMBOA (13%) was excreted unmodified (Figure 1B). Another major compound, MBOA-Glc (13%), resulted from *N*-glucosylation of MBOA, a spontaneous degradation product of DIMBOA (Figure 1A). Reduction of DIMBOA to the less toxic HMBOA (Escobar et al., 1999) appears to be an additional strategy of FAW to diminish DIMBOA toxicity. MBOA fed in sugary droplets was excreted almost exclusively as its glucoside MBOA-Glc (Figure 1C), an MBOA detoxification product in several lepidopterans (Maag et al., 2014). Ingested (2*R*)-DIMBOA-Glc was excreted mostly unchanged (Figure 1D). That is, no (2*S*)-DIMBOA-Glc was observed, suggesting, as previously reported (Glauser et al., 2011), that hydrolysis by insect enzymes to form DIMBOA aglucone was very limited and re-glucosylation did not play an important role in this context.

The excretion of the glucosylated BXD detoxification products by *S. frugiperda* at least partially involved their transport through the insect hemolymph. The presence of (2*S*)-DIMBOA-Glc and (2*S*)-HMBOA-Glc (but not the aglucones and (2*R*) epimers) in the larval hemolymph after DIMBOA feeding (Figure 1E) suggests that the glucosides produced by the insect are actively transported out of the gut cells, where they are presumably formed, into the hemolymph. As no MBOA was detected in the hemolymph, a similar scenario can also be proposed for this aglucone. Subsequently, these products are likely excreted from the hemolymph into the hindgut lumen and defecated. Accordingly, the proportions of (2*S*)-DIMBOA-Glc, (2*S*)-HMBOA-Glc, and MBOA-Glc observed in the hemolymph (Figure 1E) roughly match their abundance in frass samples (Figure 1B).

### DIMBOA-UGT Activities Are Highest in the Insect Gut Tissue

**FIGURE 2:**
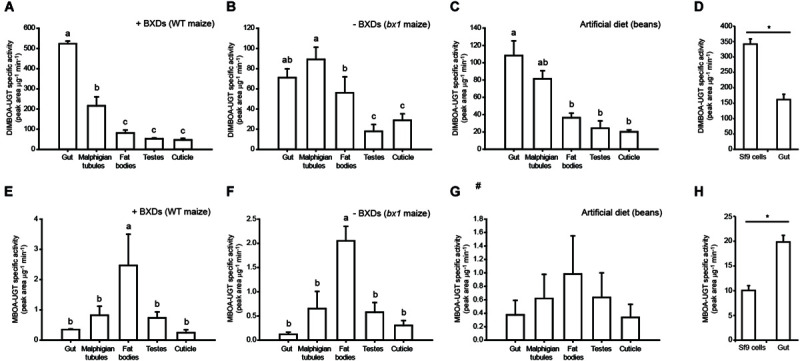
Tissue-specific profiles of UGT activity toward the benzoxazinoids DIMBOA and MBOA. BXD-UGT activity was measured towards **(A–D)** DIMBOA and **(E–H)** MBOA in *S. frugiperda* larvae feeding on WT maize leaves (BXD+), mutant *bx1* maize leaves (BXD–), and artificial diet, and in Sf9 cells (*n* = 3, mean ± SEM). One way repeated measures ANOVA was performed and the Holm–Sidak method was applied to carry out all pairwise comparisons between insect tissues. The statistical differences between individual means are denoted as small letters (a-c). *T*-tests were performed to differentiate between the means for Sf9 cells and gut tissue (*P < 0.05). #- One way repeated measures ANOVA shows no significant difference in expression levels among the tissue samples.

A comparison of *in vitro* BXD glucosylation activities among *S. frugiperda* tissues showed that DIMBOA-UGT activity was significantly higher in gut tissue than in other tissues when insects were fed on wild type maize leaves (Figure 2A), as might be expected for a detoxification enzyme (Despres et al., 2007; Ahn et al., 2012), but the differences were smaller when insects were fed on other diets (Figures 2B,C). However, somewhat unexpectedly, MBOA-UGT specific activity was significantly lower in the gut tissue than in other larval tissues, independently of the presence of BXDs in the diet (Figures 2E,F). Furthermore, the distinct profiles observed for DIMBOA- and MBOA-UGT activities among tissues indicate that these reactions involve at least two different enzymes. Surprisingly, Sf9 cells, which are derived from *S. frugiperda* ovarian tissue, were able to glucosylate DIMBOA *in vitro* more efficiently (per μg protein) than gut tissue (Figure 2D), but had lower MBOA-UGT activities than the larval tissues (Figure 2H). While it is not known how Sf9 cells have adapted to culture conditions, this has likely involved differential regulation of UGT-encoding genes that resulted in the observed activities. All *S. frugiperda* tissues, including Sf9 cells, formed exclusively the (2*S*)-DIMBOA-Glc epimer.

### Heterologously Produced UGTs Have BXD Glycosylation Activities

**FIGURE 3:**
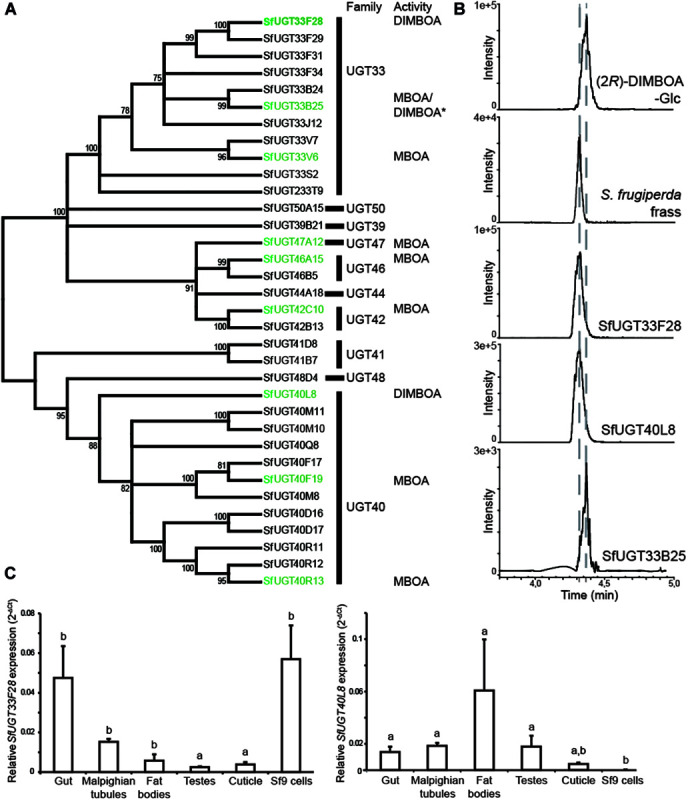
Activities of *S. frugiperda* UGTs, and expression profiles of genes encoding DIMBOA-glycosylating UGTs. **(A)** Phylogenetic tree depicting the relationships between the expressed SfUGTs and their families. Protein sequences were aligned using the Clustal algorithm and a consensus phylogenetic tree was constructed using the neighbor-joining method. Distance calculations were performed using p-distance method and bootstrap analysis. Branches corresponding to partitions reproduced in less than 70% bootstrap replicates are collapsed. The percentage of replicate trees in which the associated taxa clustered together is shown next to the branches. The UGTs found to be active toward BXDs are highlighted in green, **(B)** Schematic illustration of LC-MS/MS chromatograms from MRM analysis of DIMBOA-glucoside forming SfUGTs in enzymatic assays with DIMBOA as substrate, **(C)** Relative expression profile of genes encoding (*2S*)-DIMBOA-Glc forming UGTs (SfUGT33F28 and SfUGT40L8) measured across various *S. frugiperda* tissues and Sf9 cells by quantitative PCR (*n* = 3, mean ± SEM). One way repeated measures ANOVA was performed and Holm–Sidak method was applied to carry out all pairwise comparisons. The differences between individual means are denoted as small letters (a, b).

In order to examine which putative UGT-encoding genes are expressed in FAW larvae, we sequenced the transcriptome of larval midguts. Among 34 candidate UGT-encoding sequences, 32 full-length genes putatively encoding UGTs were successfully amplified, expressed heterologously, and the enzymes produced were screened for glucosylation activity. The sequences encoding putative UGTs obtained from our transcriptome matched well those in the whole genome published recently (Gouin et al., 2017), and the phylogenetic relationships between the successfully produced SfUGTs (*S. frugiperda* UGTs) are presented in Figure 3A. As in *H. armigera* and *B. mori*, the UGT33 and UGT40 families comprised most of the UGTs predicted in *S. frugiperda*. The recombinant SfUGTs were screened for *in vitro* activity toward general phenolic substrates (4-nitrophenol and 1-naphthol) and two BXDs (MBOA and DIMBOA) using UDP-Glc as sugar donor. When comparing the activities of different UGTs toward individual BXD substrates, protein amounts were measured and adjusted according to their relative abundances in western blots (Krempl et al., 2016). Several SfUGTs distributed among five insect UGT families, UGT33, 40, 42, 46, and 47, were active toward DIMBOA and/or MBOA (Figure 3A). Alignment of the sequences of these BXD-active SfUGTs with selected representative insect and mammalian UGTs reveals an expected conservation of important enzyme features (Supplementary Figure S2), including an N-terminal signal peptide directing translocation to the ER and substrate binding and catalytic residues. Only three (SfUGT33F28, SfUGT33B25, and SfUGT40L8) of the 32 tested UGTs accepted DIMBOA as a substrate *in vitro*. SfUGT33B25 glucosylated both DIMBOA and MBOA, but formed (2*R*)-DIMBOA-Glc, which is not the epimer present in insect frass (Figure 3B). Only SfUGT33F28 and SfUGT40L8 formed (2*S*)-DIMBOA-Glc (Figure 3B), the detoxification epimer observed *in vivo*. However, when comparing enzyme primary sequences, no single residues could be identified as probably responsible for the formation of (2*S*) instead of (2*R*)*-*DIMBOA-Glc. SfUGT33F28 and SfUGT40L8 also catalyzed the glucosylation of HMBOA yielding (2*S*)-HMBOA-Glc (Supplementary Figure S3A), another product of DIMBOA metabolism by *S. frugiperda* (Figures 1A,B), but did not glucosylate MBOA *in vitro*. This suggests that insect UGTs might be active toward BXD aglucones with similar core structures, but retain some substrate specificity. These UGTs were further tested with a small range of structurally diverse substrates, with SfUGT33F28 and SfUGT40L8 displaying weak additional activity toward some of the phenolic compounds (Supplementary Figure S4). SfUGT33B25, on the other hand, showed higher activity than SfUGT33F28 and SfUGT40L8 toward many of the compounds tested.

MBOA glucosylation is interesting as it produces an *N*-glycoside. Human UGTs have clear preferences toward either *O*- or *N*-glucuronidation, with several enzymes considered to play an important role in *N*-glucuronidation of xenobiotics (Kaivosaari et al., 2011). As mentioned earlier, the alignment of UGT protein sequences had revealed several conserved enzyme features, including the N-terminal catalytic histidine, which is important for activity toward phenolics and primary amines. Human UGTs such as UGT2B10, however, carry a leucine substitution and seem to be specialized for *N*-glucuronidation. In order to determine if the conserved histidine played a role in dictating the preference for *O*-glucosylation over *N*-glucosylation, site-directed mutagenesis of this residue was carried out. Mutation of the histidine residue to leucine in SfUGT33F28 led to largely reduced activity toward HMBOA and complete abolishment of activity toward DIMBOA (*O*-glucosylation). The mutant enzyme also did not show any gain of activity toward MBOA (*N*-glucosylation; Supplementary Figures S3B–D). However, MBOA was glucosylated *in vitro* by 6 other SfUGTs (UGT42C10, UGT40R13, UGT46A15, UGT47A12, UGT4019, and UGT33V6) belonging to several families, and none of these enzymes used DIMBOA as a substrate. While the abundance of activities observed *in vitro* might suggest a reaction with lesser specificity, it is currently not known which of these enzymes might account for the formation of MBOA-Glc *in vivo*.

### Genes Encoding DIMBOA-UGTs Are Differentially Expressed in Larval Tissues

The highest expression of the gene encoding the DIMBOA-glucosylating enzyme SfUGT33F28 (*SfUGT33F28*) was observed in the gut epithelium (Figure 3C), and its expression in different larval tissues and insect-derived Sf9 cells correlated well with the DIMBOA-UGT specific activities measured (Figure 2A). *SfUGT33F28* was the second most highly expressed UGT-encoding gene in Sf9 cells, and had by far the highest expression among genes encoding enzymes with activity toward BXDs (Supplementary Table S1, Shu et al., 2017). Expression of *SfUGT40L8*, the gene encoding the other DIMBOA 2*S*-glycosylating enzyme, however, was very low in gut tissues but high in fat bodies (Figure 3C). *SfUGT40L8* was also very low expressed in Sf9 cells, which have high DIMBOA-UGT activity (Figure 2A). These correlations between tissue-specific gene expression levels and DIMBOA-UGT activities indicate that SfUGT33F28 likely plays a more prominent role than SfUGT40L8 in DIMBOA metabolism *in vivo*. Expression of the gene encoding SfUGT33B25, which forms (2*R*)-DIMBOA-Glc *in vitro*, was highest in testes and fat bodies (Supplementary Figure S5A), which further supports its lack of involvement in BXD detoxification. Expression of selected genes encoding potential MBOA-glucosylating enzymes (Supplementary Figures S5A–D) did not match the broad patterns observed for the corresponding tissue-specific activity, so it remains unclear which enzymes are responsible for the widely distributed MBOA-UGT activity *in vivo*.

### DIMBOA-UGT-Encoding Genes and Associated Metabolic Response Are Conserved Across FAW Strains and *Spodoptera* Species

In order to examine how DIMBOA glucosylation might help FAW colonize maize plants, we compared the expression of *SfUGT33F28* in two host-differentiated *S. frugiperda* strains (corn and rice). *SfUGT33F28* expression was noted to be fairly constant across the actively feeding larval stages of the insect (Supplementary Figure S6A). When feeding on a bean-based artificial diet lacking BXDs, constitutive expression of *SfUGT33F28* was 40% higher in larvae of the corn strain than in rice strain larvae (Supplementary Figure S6B). Following a shift to maize plants, *SfUGT33F28* expression was induced 2–4 fold in larvae from both strains, with a more pronounced response in corn strain larvae (Supplementary Figure S6B). Accordingly, the DIMBOA-specific UGT activities in the midgut tissues of both strains were induced upon shifting the larvae from artificial diet to maize leaves (Supplementary Figure S6C). Furthermore, among a panel of potential UGT substrates encountered endogenously or when feeding on plant materials (selected phenolics, terpenoids, flavonoids and coumarins, in addition to BXDs), SfUGT33F28 was only able to efficiently glucosylate the BXD aglucones DIMBOA and HMBOA, with only minor activities toward some phenolic compounds also being detected (Supplementary Figure S4 and Supplementary Table S2). Therefore, although it is possible that SfUGT33F28 might have other constitutive roles *in vivo*, its apparently narrow substrate range and the inducibility of *SfUGT33F28* expression when feeding on maize leaves support an important role in BXD metabolism.

To investigate whether the role of SfUGT33F28 in BXD glucosylation is conserved among *Spodoptera* species, we investigated the closest homolog of SfUGT33F28 in *S. littoralis* (called SlUGT33F28 for convenience). Two clones encoding SlUGT33F28 with slight but consistent sequence differences were obtained (called SlUGT33F28a and SlUGT33F28b respectively), and thus both were further examined. The corn and rice strain-derived SfUGT33F28 enzymes were nearly identical to each other (2 amino acid differences along 524 residues), while the primary sequence of *S. littoralis* homologs was 92.5% identical to the corn strain SfUGT33F28 (Supplementary Figure S7). The two *S. littoralis* homologs produced heterologously also glucosylated DIMBOA efficiently, at levels similar to SfUGT33F28 (Supplementary Figure S6D). These results therefore support the conservation of enzyme function across *Spodoptera* species.

### RNAi-Mediated Knock-Down of *SfUGT33F28* Expression Reduces DIMBOA-UGT Activity in Cultured Sf9 Cells

**FIGURE 4:**
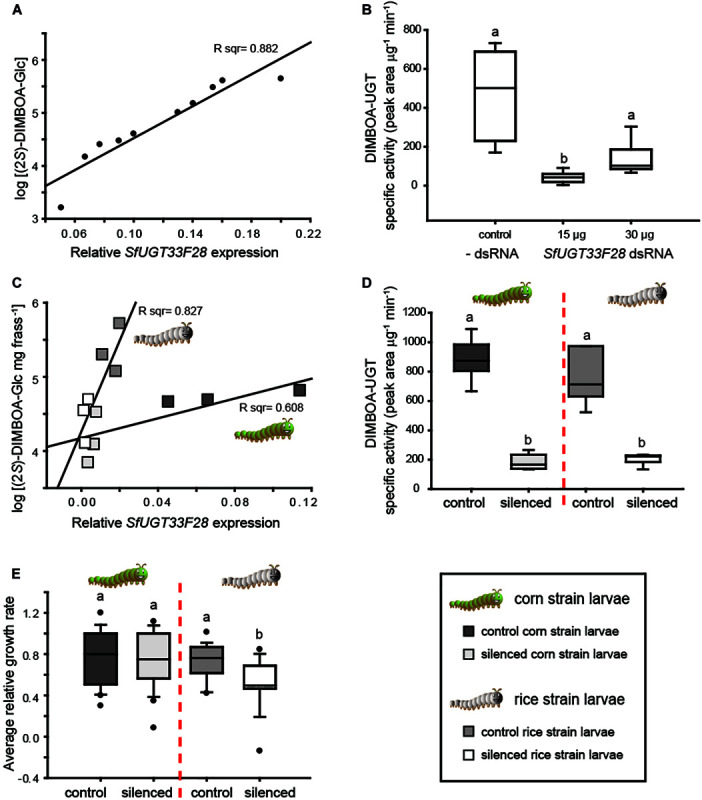
RNAi-mediated knock-down of *SfUGT33F28* in cultured Sf9 cells and *S. frugiperda* caterpillars. **(A)** Regression analysis showing the relationship between *SfUGT33F28* transcript levels and (2*S*)-DIMBOA-Glc formation in cultured Sf9 cells treated with varying concentrations of dsRNA targeting *SfUGT33F28*; **(B)** *in vitro* enzyme activities toward DIMBOA in Sf9 cell extracts; **(C)** relationship of *SfUGT33F28* transcript levels with (2*S*)-DIMBOA-Glc excretion in frass of control and *SfUGT33F28*-silenced corn and rice strain larvae; **(D)** *in vitro* enzyme activities in midguts dissected from corn strain and rice strain larvae fed with dsRNA targeting *SfUGT33F28* and **(E)** growth of corn and rice strain control and *SfUGT33F28*-silenced caterpillars on maize plants, expressed as relative growth rates [larval weight gain/(larval mean weight × feeding time)]. Data are represented as mean ± SEM; *n* = 3–5 for RNAi treatments in Sf9 cells and caterpillars, *n* = 15–20 for each biological replicate for performance assays. One way ANOVA was performed and Tukey’s test was applied for all pairwise comparisons **(B)**. Two way ANOVA was performed and Bonferroni correction was applied to differentiate between individual means **(D,E)**. The differences between individual means are denoted as small letters (a, b).

In order to further demonstrate the involvement of SfUGT33F28 in BXD detoxification, we carried out loss-of-function studies using RNAi. This was initially performed in the well-established *S. frugiperda* ovary-derived cultured Sf9 cells, which efficiently form the (2*S*)-glucoside of DIMBOA (Figure 2A). dsRNA targeting *SfUGT33F28* was transcribed *in vitro* and used according to an available protocol (Wu et al., 2016), with *SfGAPDH* serving as a transfection standard (Supplementary Figures S8A,B). Efficient (>50%) silencing of *SfUGT33F28* expression was obtained 48 h after transfection of Sf9 cells with 15 μg dsRNA and 30 μg dsRNA per 3 ml cell culture, while no change in gene expression was observed in cells transfected with transfection reagent alone or with an unrelated dsRNA control (Supplementary Figure S8C). A clear association could be observed between *SfUGT33F28* expression and (2*S*)-DIMBOA-Glc formation in the insect cells (Figure 4A). Silencing of *SfUGT33F28* led to a 5–10 fold reduction in the formation of DIMBOA-Glc (Figure 4B). No change in the expression of *SfUGT40L8*, which encodes the other enzyme capable of producing (2*S*)-DIMBOA-Glc *in vitro*, was seen in dsRNA-treated Sf9 cultures in comparison to the control cells (Supplementary Figure S8D), indicating the absence of its co-silencing or functional compensation during *SfUGT33F28* silencing. Morphological changes (increased granularity and cell shrinkage, Supplementary Figure S8E) were observed in *SfUGT33F28*-silenced Sf9 cells, which may indicate cell apoptosis (Saleh et al., 2013), and cytotoxicity measurements showed a slightly lower cell viability in *SfUGT33F28*-silenced cells (Supplementary Figure S8F). In previous studies in mammalian cells, dsRNA activated the dsRNA-dependent protein kinase, thus inactivating the translation factor eIF2α and leading to repression of protein synthesis and to apoptosis (Clemens and Elia, 1997), which can help explain the altered cell morphologies we observed. Nevertheless, in spite of possible undesirable effects, silencing of *SfUGT33F28* in cultured Sf9 cells was efficient and led to a dramatic reduction in the ability of these cells to glucosylate DIMBOA.

### *SfUGT33F28* Silencing *in vivo* Impacts BXD Detoxification and Larval Performance

After successful *SfUGT33F28* knock-down in Sf9 cells, the expression of this gene was manipulated in FAW larvae. As the *SfUGT33F28* gene is most highly expressed in gut tissues (Figure 3C), oral delivery of dsRNA was used to promote RNAi, as it is less invasive than larval microinjection. Artificial diet and maize leaves coated with bacterially produced dsRNA targeting *SfUGT33F28* were fed to FAW larvae. Gene silencing was well correlated with reduction in (2*S*)-DIMBOA-Glc accumulation in frass of dsRNA fed insects (Figure 4C). The reduction in (2*S*)-DIMBOA-Glc accumulation in the frass of silenced insects was corroborated by decreased DIMBOA-UGT enzyme activities in dissected midgut tissues (Figure 4D). The relative growth rate (RGR) of the caterpillars was also affected by *SfUGT33F28* silencing when feeding on maize leaves. However, while *SfUGT33F28* silencing led to lower (2*S*)-DIMBOA-Glc excretion in both FAW strains, the reduction of RGR was only statistically significant in the rice strain (Figure 4E). That is, although *SfUGT33F28* down-regulation was successful in both strains, its effects were variable, potentially owing to the transient nature of this silencing method, or to the existence of other adaptations to dietary BXDs in corn strain FAW larvae. Nevertheless, *SfUGT33F28* silencing led to a reduction in DIMBOA glycosylation, demonstrating the role of the SfUGT33F28 enzyme in DIMBOA glycosylation *in vivo*.

## Discussion

UDP-glycosyltransferase play crucial roles in varied aspects of insect physiology. One of the important functions of this diverse class of enzymes is in xenobiotic detoxification and plant defense modulation. A particularly interesting case is the role of glucosylation in *S. frugiperda*, an insect that thrives on members of Poaceae. Crops in the grass family, such as maize, wheat, and rye, release toxic BXD aglucones as chemical defenses upon herbivory. Previous studies have shown that FAW can glucosylate BXD aglucones such as DIMBOA by stereoselective re-glucosylation via insect UGT(s), forming products that cannot be further activated into toxic aglucones (Wouters et al., 2014). Our study takes a step further in understanding the dynamics of this selective glucosylation and identifying the underlying FAW enzyme responsible for this transformation.

As a prelude to our investigation, we performed *in vivo* feeding experiments with selected purified BXDs to gain insights into their metabolism in the insect. These assays demonstrated that FAW larvae partially metabolize DIMBOA by reducing it to the less toxic lactam HMBOA and via spontaneous degradation to MBOA in the insect gut, accompanied by glucosylation reactions that yielded (2*S*)-DIMBOA-Glc, (2*S*)-HMBOA-Glc, and MBOA-Glc respectively as major excreted products (Figure 1). BXD hydroxamic acids such as DIMBOA spontaneously degrade to benzoxazolinones in aqueous media, particularly at high pH values (Niemeyer, 2009) such as those in the FAW gut contents (Wouters et al., 2014), but the excretion of unmodified DIMBOA shows that this reaction is not quantitative in the time scale of digestion in these larvae. Very low amounts of HMBOA and MBOA aglucones were detected, suggesting that the respective reduction and spontaneous degradation reactions of DIMBOA to form these compounds are slower than the corresponding glucosylation reactions. We also detected these insect-derived BXD glucosides in hemolymph up to 6 h post ingestion, suggestive of a path of transport for their excretion. The time needed for BXD disappearance from the *S. frugiperda* hemolymph, approximately 6 h, is similar to that observed for *O. nubilalis* after topical application of ^3^H-labeled DIMBOA (Campos et al., 1989).

The profiles of UGT activities obtained from *in vitro* assays with different insect tissues revealed that the activity toward DIMBOA was strongly localized within the insect gut. This activity was increased in larvae feeding on wild type maize plants (+BXD) compared with those feeding on *bx1* mutant (−BXD) plants (Figure 2), although the +BXD and −BXD maize varieties had different genetic backgrounds and other phytochemical differences might have contributed to the higher DIMBOA glucosylation activity observed. The lower levels of UGT activity toward MBOA in gut tissues, however, were not completely understood. While fat bodies and Malpighian tubules often contribute to xenobiotic metabolism (Dow and Davies, 2006; Petersen et al., 2001; Chahine and O’Donnell, 2011), this might suggest a more general involvement of the UGTs capable of metabolizing MBOA in other constitutive functions in the insect, such as cuticle formation or pigmentation. Alternatively, since MBOA is much more lipophilic than DIMBOA, its accumulation in gut cells might be lower and it could diffuse more easily into the fat bodies, where it is detoxified. Such a spatial restriction of metabolism can help metabolites to play additional biological roles, for example the use of MBOA-Glc by *D. v. virgifera* to deter natural enemies (Robert et al., 2017). However, the absence of detectable BXD aglucones in the hemolymph of BXD-fed FAW suggests these are all similarly metabolized before leaving the gut cells in this species.

While the *in vivo* feeding experiments and *in vitro* activity tests clearly suggested a UGT-mediated glucosylation being involved in BXD detoxification, and such activities had also been proposed in the past (reviewed in Wouters et al., 2016a) little is known about the specific enzymes responsible for this conjugation. Here, we identified several UGT-encoding genes potentially responsible for these activities. Heterologous production of the corresponding enzymes revealed several UGTs with activities toward BXDs. Alignment of these BXD-active UGTs with other characterized UGTs (including a mammalian UGT) showed the conservation of amino acid residues critical for sugar donor binding and catalytic residues, with the C-terminal domain presenting the conserved UGT signature motif sequence structure [FVA]-[LIVMF]-[TS]-[HQ]-[SGAC]-G-x(2)-[STG]-x(2)-[DE]-x(6)-P-[LIVMFA]-[LIVMFA]-x(2)-P-[LMVFIQ]-x(2)-[DE]-Q (Meech and Mackenzie, 1997). The N-terminal region which is likely responsible for binding the aglucone is less conserved than the C-terminal region which is thought to bind the UDP-sugar. The low degree of conservation in the N-terminal region between the members of this family is a feature that is shared with UGT members in many other organisms as aglucones bound by UGTs can be highly diverse (Mackenzie, 1990; Huang et al., 2008).

While several UGTs had variable activity toward the major maize BXDs, SfUGT33F28, a member of the UGT33 superfamily, was found to be rather specific toward DIMBOA among the substrates tested. *SfUGT33F28* was highly expressed in the insect gut, in accordance with the high DIMBOA-UGT activity observed in this tissue (Figure 3). Furthermore, the conservation of DIMBOA glucosylation activity in the closest homolog of SfUGT33F28 in another *Spodoptera* species suggests that this pathway might have a role in the adaptation of these insects to BXD-producing plants. The differences in glucosylation between the two *S. littoralis* clones were not expected, as the residues deemed important for catalysis were conserved, and might stem from changes in a few amino acid residues with unexplored implications for substrate/acceptor binding, optimal folding and consequently enzyme activity. For instance, substitution of an aspartate (D336) within the UGT signature motif of SlUGT33F28b by the non-polar alanine in SlUGT33F28a might have implications for enzyme function. A similar replacement of negatively charged aspartate (D357A) and glutamate (E378A) residues in human UGT1A6 with alanine has been shown to reduce UGT activity by 3.2 fold and 150 fold respectively, and lowered the affinity for the substrate (Li et al., 2007). Nevertheless, taken together, these results support the conservation of enzyme function across *Spodoptera* spp., but suggest that SfUGT33F28 itself may not be particularly adapted or optimized toward the effective use of DIMBOA as a substrate, with other adaptations likely being used in concert with plant defense detoxification when attacking maize plants. On the other hand, we observed constitutive differences in *SfUGT33F28* expression and inducibility across different strains, as well as variable responses to *SfUGT33F28* knock-down, as evidenced by the differences in the growth of silenced rice strain caterpillars over silenced corn strain caterpillars (Figure 4). It is, however, still unclear what additional factors may underlie these differences between strains, which ultimately affect their association with their host plants. Although the FAW strains show similar patterns of leaf consumption (Veenstra et al., 1995) and both strains have been frequently sampled from maize fields (Meagher and Nagoshi, 2004; Nagoshi et al., 2007), they may nevertheless differ in their degrees of elicitation and susceptibility to host plant defenses, including those other than BXDs (Acevedo et al., 2017, 2018). Additionally, although FAW from different geographical origins have been shown to differ for example in resistance to *Bacillus thuringiensis* toxins and in responses to pheromones (Farias et al., 2014; Unbehend et al., 2014), it is unknown whether they might also differ in BXD metabolism or susceptibility, or in the plant responses they elicit, and how well the two strains tested here might represent such variation. Furthermore, diversity in insect UGTs can be generated by alternate splicing events (Ahn et al., 2012), but the resulting effects are often not immediately apparent and may gain importance only when functional gene copies are eliminated. This kind of functional redundancy is a common feature of detoxification gene families (Wagner, 1996; Feyereisen, 1999) and might represent another stratum of strain-specific gene regulation in the UGT superfamily.

In conclusion, our results show that DIMBOA ingested by FAW when feeding on maize leaves is transformed via different pathways, with glucosylated products representing the majority of excreted BXDs. The UGT33 family member SfUGT33F28 seems to be responsible for the detoxification of the major maize defensive compound DIMBOA, but not for the metabolism of the also abundant benzoxazolinone MBOA. This detoxification reaction, along with other potential adaptations for feeding on grass crops, might contribute to the current violent global spread of this pest on maize crops.

## Materials and Methods

### Insects and Plants

Larvae of *S. frugiperda* were maintained at the Department of Entomology of the Max Planck Institute for Chemical Ecology. Two host differentiated strains of *S. frugiperda* were used in the present study, Puerto Rico corn strain (prc) and Florida rice strain (flr). These populations were established post screening for strain-specific cytochrome oxidase I gene (COI) markers (Nagoshi et al., 2006; Hänniger et al., 2017). Larval instars used for different assay purposes were chosen according to ease of handling (2nd instar for performance experiments, as the daily consumption by these larvae ensured a constant food supply, and 3rd–5th instar for collection of frass and dissection of larval tissues due to their larger sizes). Eggs of *S. littoralis* were a generous gift from Syngenta Crop Protection (Stein, Switzerland). *S. frugiperda* was reared on artificial diet based on pinto bean, while *S. littoralis* was reared on an artificial diet based on white bean. All insects were maintained under controlled light and temperature conditions (12:12 h light/dark, 21°C). Seeds of *Zea mays* were obtained commercially (Badischer Gelber variety, Kiepenkerl, Germany), seeds of *bx1* mutant were obtained from the Maize Genetics Cooperation Stock Center of the USDA/ARS at the University of Illinois, Urbana/Champaign^1^ (stock 428G) and grown under controlled light and temperature conditions (16:8 h light/dark, day-time temperature 22°C, night-time 20°C).

### Droplet Feeding Assays

Individual solutions containing 10 mM of DIMBOA, 5 mM of MBOA, 20 mM of (2*R*)-DIMBOA-Glc, or 20 mM (2*R*)-HMBOA-Glc were prepared by dissolving the BXDs in DMSO (5% of the final volume) and diluting them in 10% sucrose solution in water. Fourth to fifth instar *S. frugiperda* larvae were starved for 24 h, stimulated with forceps immediately before the feeding in order to regurgitate, and dried with paper towel. With the assistance of forceps and a microscope, BXD solutions (2 μL) were administered directly to larval mouthparts using a micropipette. Approximately half of the individuals used were receptive to droplet feeding, by actively and completely sucking the BXD solution from the pipette tip. Individuals that regurgitated or moved causing losses of the solution during the feeding were discarded. Larvae used for frass collection were then left to feed individually on artificial diets and kept under the regular rearing conditions for 24 h. After this time, the frass samples in the plastic cups were collected thoroughly, and extracted with 1 mL of a water:methanol 1:1 (v:v) solution. After adding 3 mm steel beads, extracts were vortexed for 2 min, agitated in a paint shaker for 2 min, and centrifuged at 16,000 *g* for 5 min. This extraction was repeated two more times and the combined supernatants were dried in a centrifugal vacuum concentrator (Eppendorf Concentrator 5301, Eppendorf, Hamburg, Germany), re-suspended in 200 μL of 0.5% formic acid in water:methanol 1:1 (v:v) solution, and analyzed by LC-MS/MS. Droplet samples (2 μL) from the administered solutions were diluted and quantified by LC-MS/MS in order to normalize the ingested amount of each BXD.

Individuals for hemolymph collection were kept feeding on artificial diet on the bench after droplet feeding. After 1, 2, 3, 6, and 12 h, individuals (*N* = 3) were anesthetized by −20°C treatment for 5 min, and had one proleg severed with dissecting scissors without damaging the gut tissue. With a micropipette, 10 μL of the hemolymph droplet were collected and immediately transferred to a tube containing 40 μL of 0.5% formic acid in water:methanol 1:1 (v:v) solution on ice. The resulting samples were centrifuged at 5,000 *g* for 5 min and the supernatant was collected and analyzed by LC-MS/MS.

### Insect Cell Cultures

*Spodoptera frugiperda* Sf9 cells and *Trichoplusia ni* High Five cells were cultured in Gibco Sf-900 II serum-free medium (Thermo Fisher Scientific, United States) and Gibco ExpressFive serum-free medium (Thermo Fisher Scientific, United States), respectively. Adherent cultures were maintained at 27°C, and sub-cultured every 3–4 days.

### RNA Extraction and Reverse Transcription

For transcriptome sequencing, third to fourth instar larvae of *S. frugiperda* were fed maize leaves (2 weeks old plants, L4 stage) for 48 h, followed by dissection on cold phosphate buffer (pH 7.0, 10 mM). Guts and integument were collected separately, gut lumen briefly washed and stored in RNAlater (Sigma-Aldrich, Germany) overnight at 4°C before RNA extraction. Tissue samples from two individuals were pooled together for RNA extraction. Samples of Sf9 cell cultures (obtained during sub-culturing at full confluency) were centrifuged at 500 *g* for 5 min, the culture medium was discarded, and the fresh pellets were directly used for RNA extraction. Total RNA was extracted from tissues and Sf9 cell samples using the innuPREP RNA Mini Kit (Analytik Jena, Germany), followed by DNase treatment. RNA concentrations were measured with the NanoDrop 2000 UV-Vis Spectrophotometer (Thermo Scientific). First Strand cDNA was synthesized from 1 μg total RNA using SuperScript III Reverse Transcriptase and OligodT primers from Invitrogen.

### cDNA Library Construction and Illumina Sequencing

cDNA was prepared from total RNA using NEBNext Ultra Directional RNA Library Prep Kit for Illumina (New England Biolabs) according to the manufacturer’s instructions. Library construction and sequencing were performed by the Max Planck Genome Center Cologne, Germany^2^ using the manufacturers’ protocols. TruSeq libraries were generated from poly-A enriched mRNA. The library was sequenced with an Illumina HiSeq2500 sequencer, and after de-multiplexing 8–11 million 100 bp paired-end reads per sample were obtained. The resulting sequencing data can be freely accessed at doi: 10.17617/3.55. Short 100 bp paired-end Illumina reads were quality-trimmed and filtered for duplicates using default parameters and then assembled *de novo* using the CLC Genomics Workbench software package (CLC bio Qiagen), using bubble size = 65. Consensus sequences were extracted to give 49,836 contigs.

### Bioinformatics Analysis

Contigs obtained from RNA-Seq analyses were used as a database for tblastx queries from *H. armigera* UDP-glycosyltransferase sequences (Ahn et al., 2012). The resultant 63 contigs were considered putative *S. frugiperda* UGTs, whose sequences were further assembled using the short Illumina reads until they reached the expected size for an insect UGT (1500–1600 bp) and resemblance to described *H. armigera* UGT domains. After discarding redundant contigs and sequences that did not show the typical UGT structure, the resulting full sequences were confirmed by comparison with the published *S. frugiperda* draft genome (Kakumani et al., 2014) (NCBI:JQCY00000000) and transcriptome databases (Nègre et al., 2006; Legeai et al., 2014; Kakumani et al., 2015) (NCBI:GCTM00000000, Lepidodb:TR2012b^3^, and Spodobase^4^), and were used to design full sequence primers used for heterologous expression. Geneious 9.1.3 software was used for sequence data treatment.

### Cloning and Heterologous Expression

Full sequence primers were designed based on the UGT sequences retrieved from RNA-Seq analysis, covering 3–15 bases before the start codon and lacking the stop codon and obtained from Sigma-Aldrich (Supplementary Table S3). From the 34 UGT candidates with full sequences retrieved, 32 candidates were successfully amplified from *S. frugiperda* gut cDNA samples using Phusion High Fidelity DNA Polymerase (New England Biolabs) (PCR protocol: 30 s at 98°C; 35 cycles of 10 s at 98°C, 20 s at 60°C, 45 s at 72°C; and 5 min at 72°C). The resulting amplified products were purified with a PCR cleanup kit (Qiagen), and incubated with GoTaq DNA polymerase (Promega, United States) for 15 min at 72°C in order to add A overhangs. The products were directly cloned into the pIB/V5-His-TOPO (Thermo Fisher Scientific, United States) vector and transformed into TOP10 (Thermo Fisher Scientific, United States) cells, which were plated on selective LB agar medium containing 100 μg mL^–1^ ampicillin and incubated overnight at 37°C. Positive colonies were identified by PCR using vector-specific primers OpIE2, sub-cultured overnight at 37°C in liquid LB medium containing 100 μg mL^–1^ ampicillin, and used for plasmid DNA purification with the NucleoSpin Plasmid kit (Macherey-Nagel, Germany). Concentration and purity of the obtained constructs were assessed by a NanoDrop 2000 UV-Vis Spectrophotometer (Thermo Fisher Scientific, United States) and the correct orientation of the PCR products was confirmed by DNA sequencing. For site-directed mutagenesis, non-linearized pIB/V5-His TOPO-TA vector carrying *SfUGT33F28* was used as a template for PCR using mutagenic primers (Supplementary Table S4). As the template plasmid DNA was methylated during replication in *E. coli*, plasmid containing the wild type gene sequence was removed by incubation with the methylation-specific restriction enzyme *Dpn*I. To re-obtain circular plasmid from the linear PCR product, 1 μl T4 ligase enzyme and 2 μl 5x T4 ligase buffer were added to the reaction and *E. coli* cells were transformed.

For transfection, *T. ni* HighFive cells (Thermo Fisher Scientific, United States) were sub-cultured at full confluency in a 6-well plate in a 1:3 dilution, and left overnight to adhere to the flask surface. The medium was replaced and transfections were carried out using FuGENE HD Transfection reagent (Promega, United States) on a 1:3 plasmid:lipid ratio (1.7 μg plasmid and 5.0 μL lipid for 3 mL medium). Cells were incubated for 48 h at 27°C, re-suspended in fresh medium, and divided in three aliquots, which were used for re-seeding, a UGT enzymatic assay, and Western blot analysis. Re-seeded cells were selected by growing in culture medium containing 50 μg mL^–1^ blasticidin for 2 weeks and tested for UGT activity again. Transfected cells that showed activity toward DIMBOA and/or MBOA were maintained at 10 μg mL^–1^ blasticidin as stable cell cultures.

### Enzymatic Assays

For comparative UGT assays with tissues, *S. frugiperda* third to fourth instar caterpillars feeding on artificial diet were used. Caterpillars were dissected in cold phosphate buffer (pH 7.0, 10 mM), and their gut tissues and integument were collected separately, and cleaned from gut contents. Tissue samples were homogenized in the same buffer. Samples from insect cell cultures (transient or stable) were collected, centrifuged at 500 *g* for 5 min, and pellets re-suspended and homogenized in phosphate buffer (pH 7.0, 100 mM). Aliquots of these enzyme preparations were boiled for 20 min. Typical enzyme reaction included 10 μL homogenate/cell extract, 2 μL of 12.5 mM DIMBOA or MBOA in DMSO (25 nmol), 4 μL of 12.5 mM UDP-glucose in water (50 nmol), and enough phosphate buffer (pH 7.0, 100 mM) solution to give an assay volume of 50 μL. Enzymatic assays for Sf9 cells included 44 μL cell homogenate, 2 μL of 12.5 mM DIMBOA or MBOA in DMSO, and 4 μL of 12.5 mM UDP-glucose in water. Controls containing either boiled enzymatic preparation, or only the protein suspension and buffer were included. After incubation at 30°C for 60 min, the enzyme reactions were interrupted by adding 50 μL of a methanol:formic acid 1:1 (v:v) solution. Assay tubes were centrifuged at 5,000 *g* for 5 min and the obtained supernatant was collected and analyzed by LC-MS/MS.

### Real Time PCR Analysis

Real time PCR analyses were carried out using Brilliant III Ultra-Fast SYBR Green QPCR Master Mix (Agilent, United States). The real time primers used for the present study have been provided in Supplementary Table S5. Relative quantification of the transcript levels was done using the method described by Livak and Schmittgen (2001). SfRPL10 was used as reference gene for all analyses.

### *In vitro* Transcription of *SfUGT33F28* and Gene Silencing in Sf9 Cells

The SfUGT33F28 RNAi amplicon was amplified using gene specific primers carrying the T7 polymerase site (Supplementary Table S6). The PCR product thus obtained was used as a template for the *in vitro* transcription reaction. The reaction was assembled following the guidelines provided in the MEGAscript T7 *In Vitro* Transcription kit (Thermo Fisher Scientific, United States). The product obtained was quantified using Nanodrop and the integrity of the dsRNA was confirmed on a 1.2% denaturing agarose gel.

For the silencing experiments, Sf9 cells were seeded in 2 mL of Sf900 II serum free medium (SFM) in 6 well plates at 5 × 10^5^ cells mL^–1^ and allowed to grow overnight at 27°C. Meanwhile, desired dilutions of dsRNA were made in SFM (2 mL for each replicate). Transfection was carried out using FuGENE HD transfection reagent. dsRNA specific to the target gene was added to the wells and the cells were incubated for 4–6 h. A transfection control (Sf9 cells plus transfection reagent) was included to ensure that no change in gene expression and cell viability resulted upon treatment with the reagent alone. A control template provided in the kit (*Xenopus* elongation factor 1α) was used as an unrelated dsRNA control. The solution containing dsRNA was replaced with SFM and cells were allowed to grow for another 48 h at 27°C.

### 3-(4,5-Dimethylthiazol-2-yl)-2,5-Diphenyl Tetrazolium Bromide (MTT) Assay

MTT (Sigma-Aldrich, Germany) assays were performed for assessment of cell viability. About 1 mL of control and treated Sf9 cell suspensions were centrifuged at 1200 rpm for 5 min. The pellet was weighed and re-suspended in 100 μL SFM. MTT (100 μL, 1 mg mL^–1^) was added to each suspension and left for incubation for 2–4 h at 27°C, until purple insoluble formazan crystals appeared. Crystals so formed were collected by centrifugation at 1200 rpm for 5 min, and re-suspended in 200 μL DMSO. Solutions containing the suspended crystals were transferred to microtiter plates, and absorbance was measured at 570 nm. Cell viability was calculated as:

(O.D. treated cells-O.D. blank wells/O.D. control-O.D. blank wells) × 100.

### Production of *SfUGT33F28* dsRNA in Bacteria and *in vivo* Feeding Assay

L4440 vector harboring an IPTG-inducible dual T7 promoter system was employed for dsRNA synthesis in bacteria [L4440 was obtained from Andrew Fire (Addgene plasmid # 1654^5^; RRID:Addgene_1654]. The vector harboring the SfUGT33F28 RNAi amplicon was mobilized in RNase deficient One Shot BL21 star (DE3) competent cells (Invitrogen). The L4440:*SfUGT33F28* RNAi clone was cultured overnight in 3 mL LB broth. Secondary cultures were grown the subsequent day in 100 mL LB medium. After reaching an O.D. between 0.4 and 0.6, cultures were induced using 0.5 mM IPTG and the cells were allowed to grow for another few hours. Cells were harvested by centrifugation at 4500 rpm for 10 min at 4°C and the pellets were re-suspended in 1 mL of nuclease free water. The amount of dsRNA produced was quantified on 1.2% denaturing agarose gel using *in vitro* transcribed dsRNA as control. For the feeding assay, both pinto bean based artificial diet and maize leaves were used. The concentration of bacteria to be used was determined empirically. For diet based assays, diet plugs weighing 1–1.5 g were coated with 50–100 μL of the homogenized bacterial suspension. The volume of the suspension used was standardized by electrophoresis of equal amounts of the bacterial extracts and *in vitro* transcribed ds*UGT33F28*, providing a measure of the amount of RNA produced by the bacteria. Empty vector L4440 was kept as a control during the entire course of the assay. Likewise for detached leaf based assays, small leaf slices were coated with same volume of bacterial suspension. Feeding was carried out for 2 days. dsRNA coating was renewed every day (with fresh leaves in case of leaf based assays) during the course of the experiment.

### Larval Performance Assays

Performance assays were carried out using 2nd instar larvae. The insects were fed with ds*UGT33F28*-coated leaves for 4 days, with larval weights being recorded at 2 and 4 days. The insects were confined to feeding on a single leaf, which was changed every day at a fixed time. Effects on larval growth were calculated as RGR between days 2 and 4. RGR was determined as: Larval weight gain/(Larval mean weight × Feeding time).

### Chromatographic Methods

For all analytical chromatography procedures, formic acid (0.05%) in water and acetonitrile were used as mobile phases A and B, respectively, and the column temperature was maintained at 25°C. Analyses of enzymatic assays and plant samples used an XDB-C18 column (50 × 4.6 mm, 1.8 μm, Agilent Technologies, Boeblingen, Germany) with a flow rate of 1.1 mL min^–1^ and with the following elution profile: 0–0.5 min, 95% A; 0.5–6 min, 95–67.5% A; 6.02–7 min, 100% B; 7.1–9.5 min, 95% A. For enzymatic assays that formed DIMBOA-Glc, resolution between the two epimers was achieved using a Nucleodur Sphinx RP column (250 × 4.6 mm, 5 μm, Macherey-Nagel, Germany) at a flow rate of 1.0 mL min^–1^ with the following gradient: 0–10 min, 85% A; 10.1–12 min, 100% B; 12.1–15 min, 85% A. LC-MS/MS analyses were performed on an Agilent 1200 HPLC system (Agilent Technologies, Boeblingen, Germany) coupled to an API 3200 tandem spectrometer (Applied Biosystems, Darmstadt, Germany) equipped with a turbospray ion source operating in negative ionization mode. The ion spray voltage was maintained at −4500 V. The turbo gas temperature was 500°C, nebulizing gas 60 psi, curtain gas 25 psi, heating gas 60 psi and collision gas 5 psi. Multiple reaction monitoring (MRM) was used to monitor analyte parent ion to product ion conversion with parameters from the literature for DIMBOA (Pedersen et al., 2011) and DIMBOA-Glc (Wouters et al., 2014). MRM parameters were optimized from infusion experiments with standard samples for MBOA-Glc (Q1 *m/z*: 372, Q3 *m/z*: 164, DP −15 V, EP −4.5 V, CEP −18 V, CE −20 V, CXP −4 V). Both Q1 and Q3 quadrupoles were maintained at unit resolution. Absolute quantities of the measured BXDs were determined using standard curves obtained from purified samples of DIMBOA (1–100 μM), (2*R*)-DIMBOA-Glc (1–10 μM), and MBOA-Glc (0.1–5 μM). A (2*R*)-DIMBOA-Glc standard curve was used for quantification of both (*2R*) and (2*S*) epimers. Analyst 1.5 software (Applied Biosystems, Darmstadt, Germany) was used for data acquisition and processing.

### Reagents and Solvents

DIMBOA used for UGT assays and BXD standards [(2*R*)-DIMBOA-Glc and MBOA-Glc] were kindly provided by Prof. Dieter Sicker (University of Leipzig, Germany), and Dr. Gaétan Glauser (University of Neuchâtel, Switzerland) respectively. MBOA (Sigma-Aldrich, Germany) and UDP-glucose (Santa Cruz Biotechnology, Germany) were commercially obtained.

### Statistical Analysis

All statistical analyses were performed using SigmaPlot 12.0 (Systat Software Inc.). The statistical test(s) performed and the number of replicates used in each experiment are described in the figure legends.

## Data Availability Statement

The datasets presented in this study can be found in the Supplementary Material and in the online repositories listed in the article.

## Author Contributions

BI, FW, S-JA, ME, DH, JG, and DV conceived and designed the experiments. BI, FW, KL, ES, CP, and MR performed the experiments. All the authors evaluated the data and interpreted the results. BI, FW, and DV wrote the manuscript with input and approval from all authors.

## Conflict of Interest

The authors declare that the research was conducted in the absence of any commercial or financial relationships that could be construed as a potential conflict of interest.

## Footnotes

1 http://maizecoop.cropsci.uiuc.edu

2 http://mpgc.mpipz.mpg.de/

3 http://www6.inra.fr/lepidodb/Downloads/TR2012b

4 http://bioweb.ensam.inra.fr/spodobase/

5 http://n2t.net/addgene:1654
