# Supplementary material for: The Fall Armyworm Spodoptera frugiperda Utilizes Specific UDP-Glycosyltransferases to Inactivate Maize Defensive Benzoxazinoids
Source: Front Physiol. 2020 Dec 21;11:604754. doi: 10.3389/fphys.2020.604754 (PMC7781194; doi:10.3389/fphys.2020.604754)
Supplement: Supplementary file 1 [file Data_Sheet_1.PDF]

**SUPPLEMENTARY INFORMATION**

# The fall armyworm *Spodoptera frugiperda* utilizes specific UDP-glycosyltransferases to inactivate maize defensive benzoxazinoids

Bhawana Israni, Felipe C. Wouters, Katrin Luck, Elena Seibel, Seung-Joon Ahn, Christian Paetz, Maximilian Reinert, Heiko Vogel, Matthias Erb, David G. Heckel, Jonathan Gershenzon, Daniel Giddings Vassão

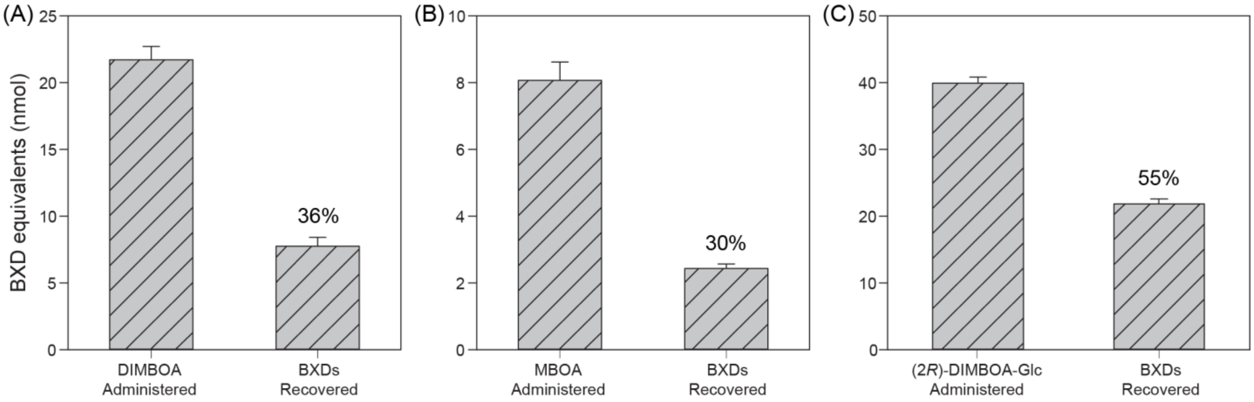


**Supplementary figure 1. Recovery of BXD metabolites in *S. frugiperda* frass.** Overall rates of recovery calculated after oral administration of DIMBOA, MBOA and (2*R*)-DIMBOA-Glc (n=5-6, mean ± SEM).

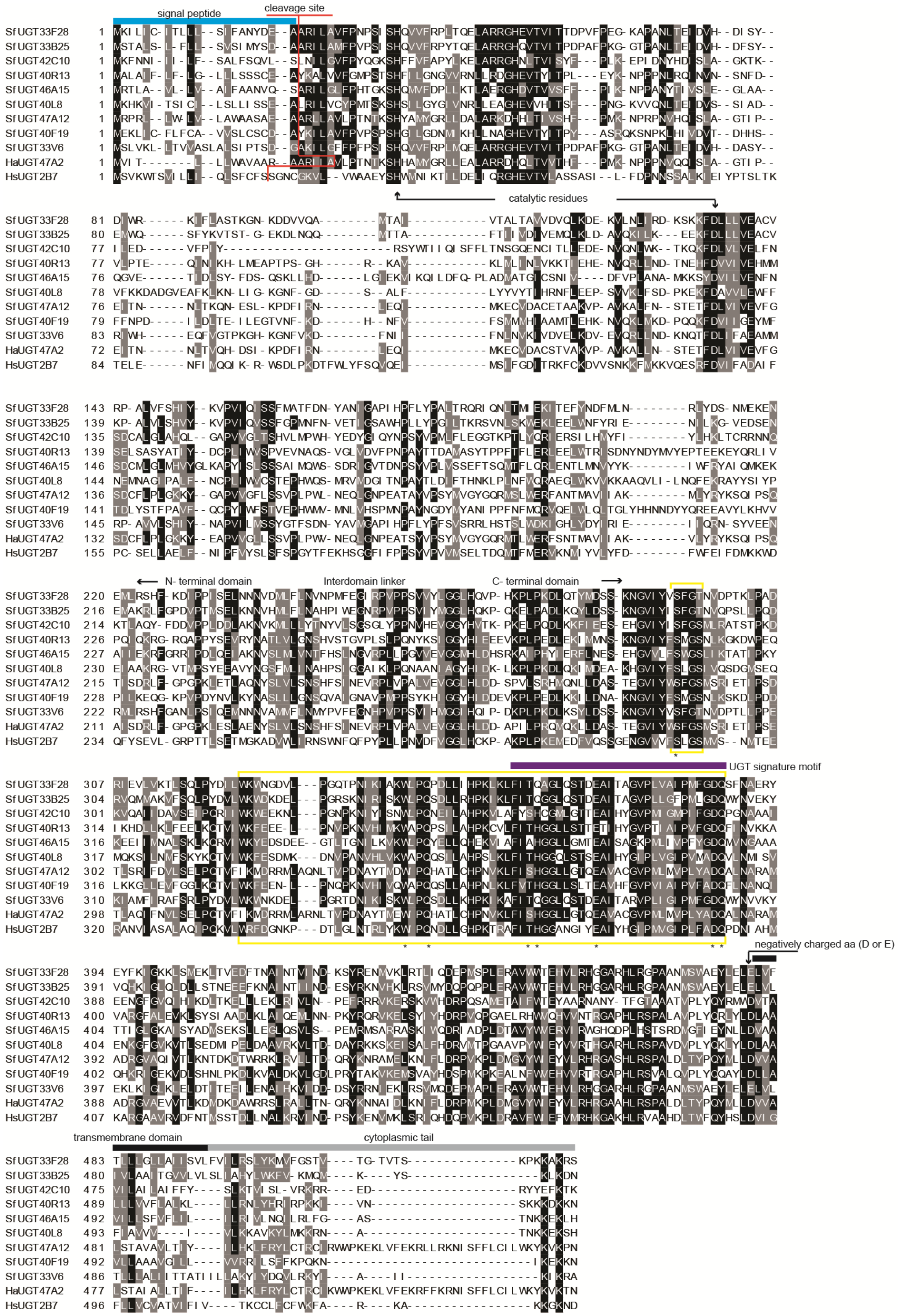
signal peptide
cleavage site
catalytic residues
N- terminal domain
Interdomain linker
C- terminal domain
UGT signature motif
negatively charged aa (D or E)
transmembrane domain
cytoplasmic tail
SfUGT33F28
SfUGT33B25
SfUGT42C10
SfUGT40R13
SfUGT46A15
SfUGT40L8
SfUGT47A12
SfUGT40F19
SfUGT33V6
HaUGT47A2
HsUGT2B7

**Supplementary figure 2. Multiple sequence alignment of functionally characterized *S. frugiperda* UGTs active towards benzoxazinoids**. The primary protein structures of SfUGTs with potential function in benzoxazinoid detoxification are compared to the human UGT2B7 and the insect UGT47A2 from *H. armigera*. The predicted signal peptide, UGT signature motif, transmembrane domain and cytoplasmic tail are shown as bars on top of the alignment. The catalytic residues, putative sheets as well as regions (donor binding regions/ DBR1 and 2, yellow box) and residues (*) responsible for sugar-donor binding in the C-terminal domain were predicted based on the crystal structure and mutational analysis reported for UGT2B7.

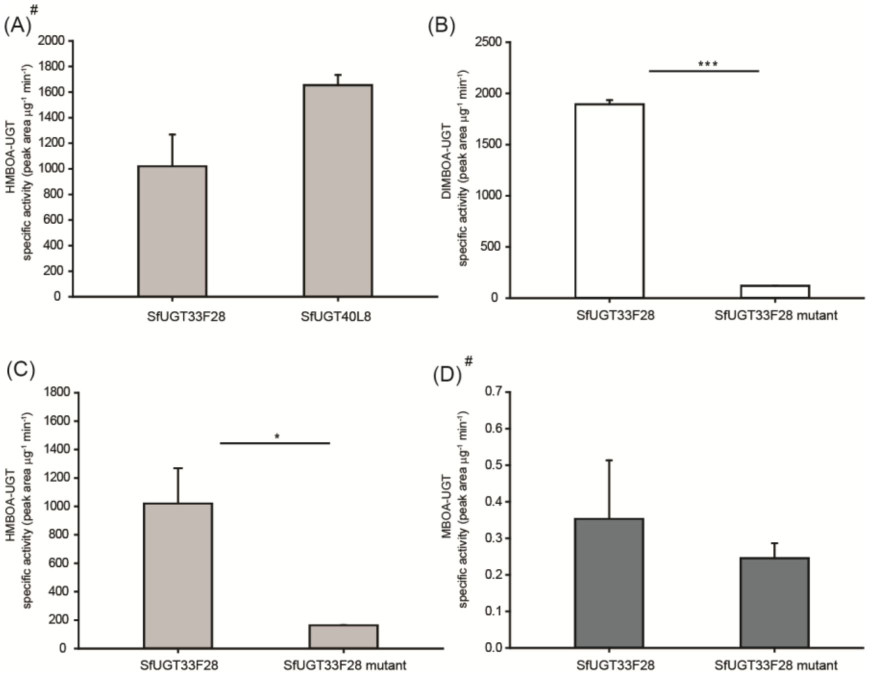


**Supplementary figure 3. Activity profiles of SfUGT33F28 and SfUGT40L8.** (A) SfUGT33F28 and SfUGT40L8 enzyme activities towards HMBOA, (B)-(D) SfUGT33F28 and SfUGT33F28-H34L site-directed mutant enzyme activities towards DIMBOA, HMBOA and MBOA respectively. Data are presented as mean ± SEM (n=3). T-tests were performed to differentiate between the means (*P < 0.05, ***P < 0.001 according to the t-test used in the present study). #- no significant difference between the means.

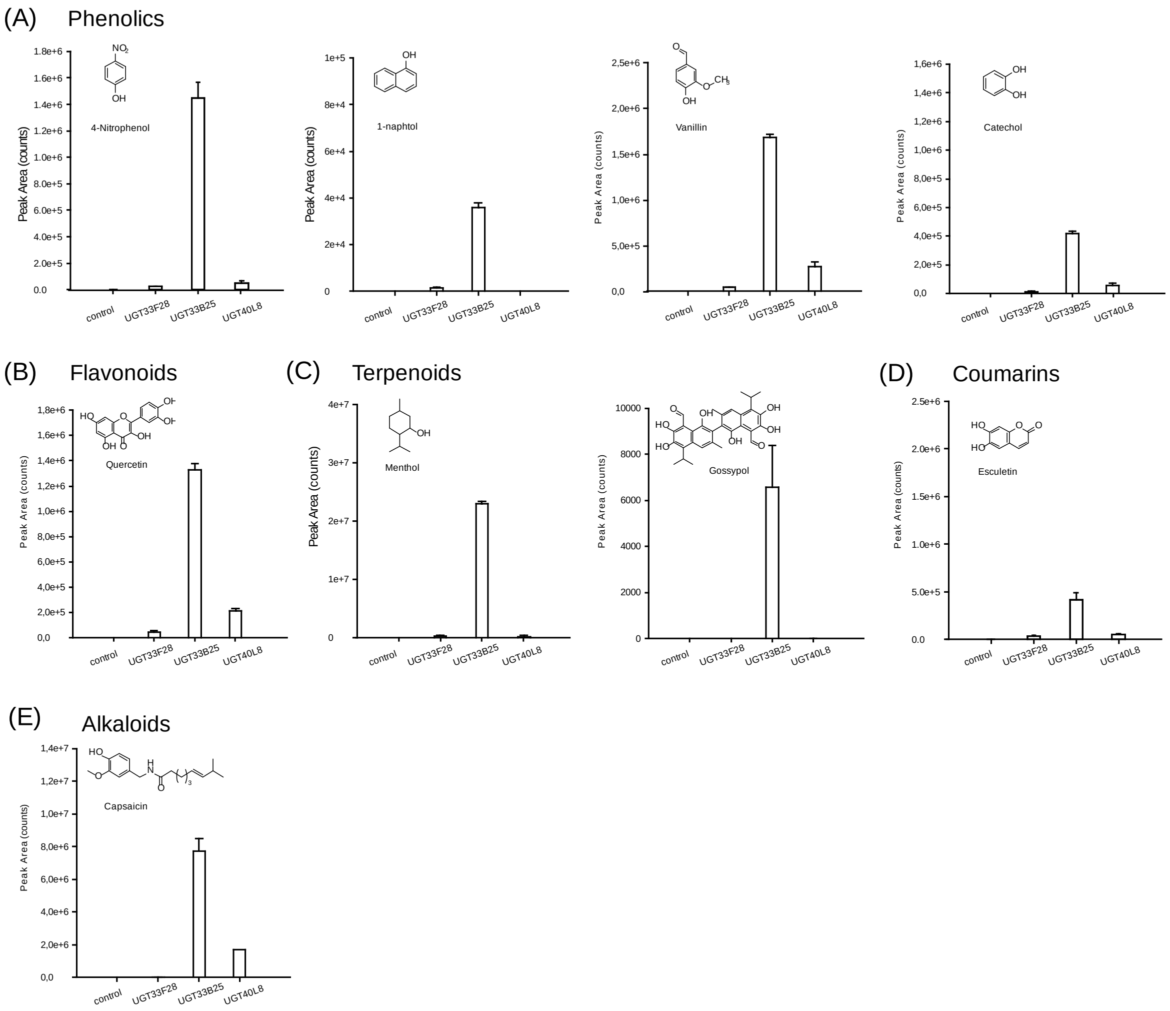


**Supplementary figure 4. Comparison of glucosylation activities of SfUGT33F28, SfUGT33B25 and SfUGT40L8 towards different classes of UGT substrates.** The three DIMBOA-active UGT enzymes were additionally tested for activities towards a small panel of structurally diverse aglucone substrates classified generally as (A) phenolics, (B) flavonoid, (C) terpenoids, (D) coumarin and (E) alkaloid. Activities are expressed as raw peak areas from extracted LC-MS chromatograms.

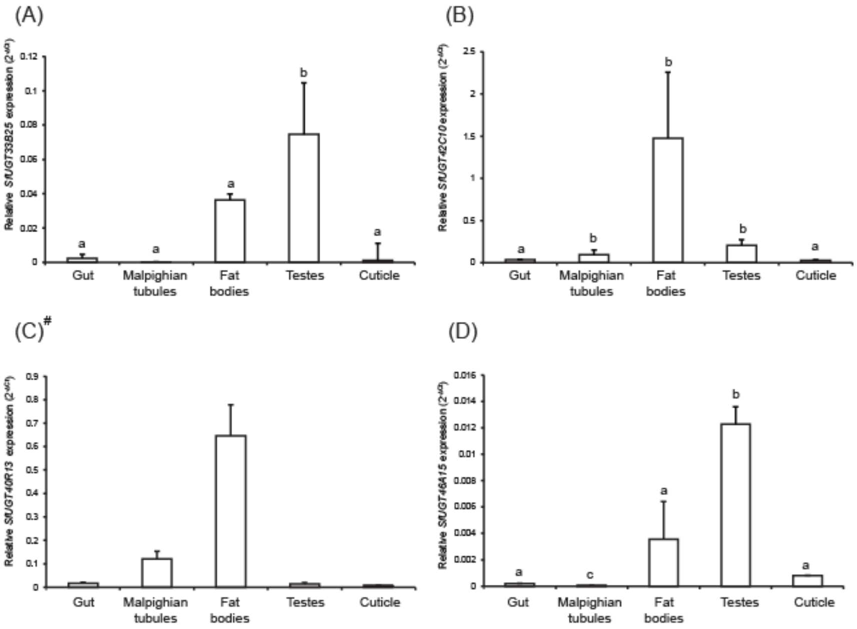


**Supplementary figure 5. Relative expression profile of genes encoding MBOA glycosylating enzymes across various *S. frugiperda* tissues**. Relative transcript abundances of *SfUGT33B25* (A), *SfUGT42C10* (B), *SfUGT40R13* (C) and *SfUGT46A15* (D) were measured across different tissues of *S. frugiperda* by quantitative PCR (n=3, mean ± SEM). One way repeated measures ANOVA was performed and Holm-Sidak method was applied to carry out all pairwise comparisons. The differences between individual means are denoted as small letters (a-c). #- One way ANOVA shows no significant difference in expression levels among the tissue samples.

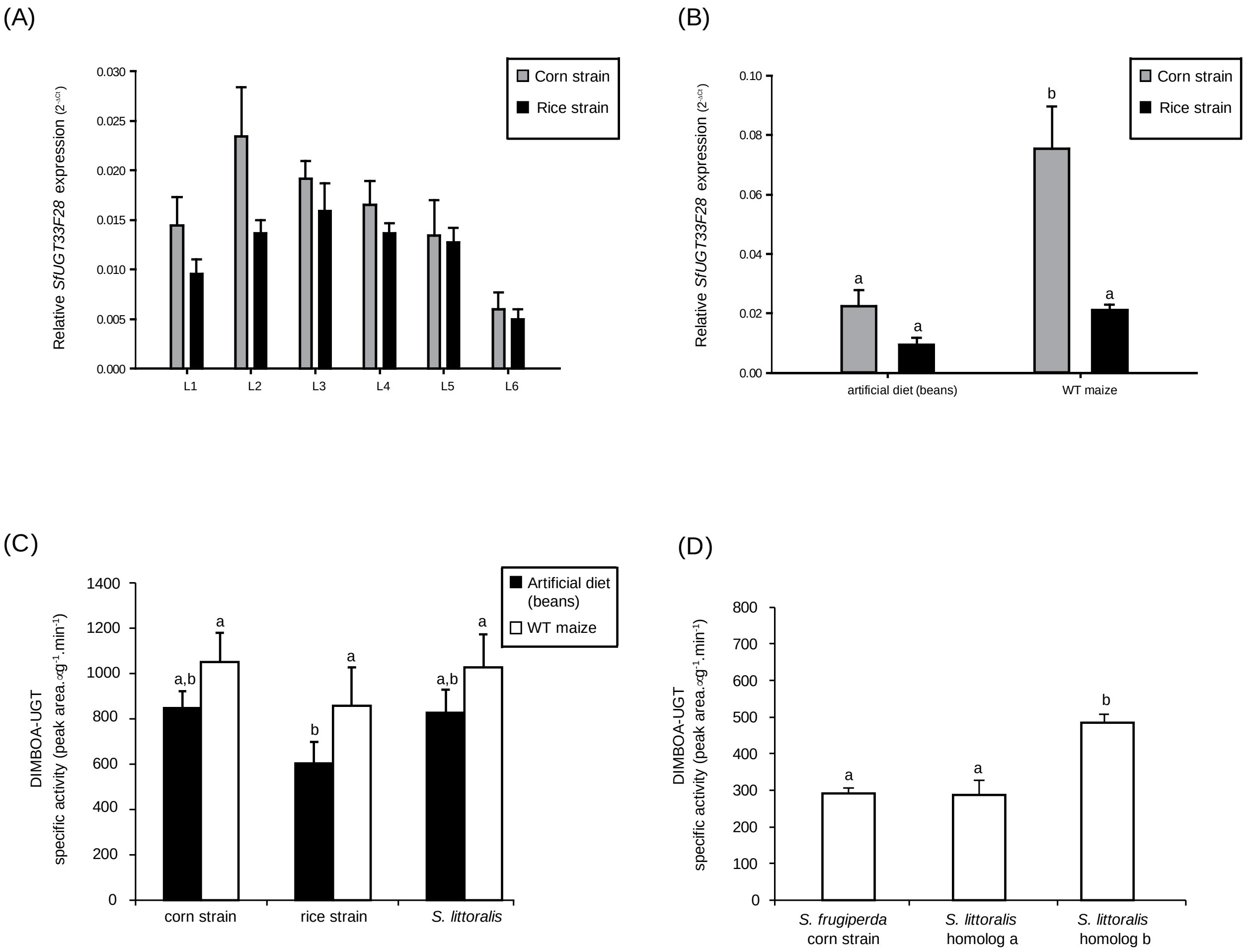


**Supplementary figure 6. Expression profile of *SfUGT33F28* and comparative account of UGT33F28 mediated glycosylation activity in two *Spodoptera* species**. (A), Relative expression levels of *SfUGT33F28* across larval stages of corn and rice strain FAW caterpillars maintained on bean-based artificial diet, (B) Relative expression levels of *SfUGT33F28* during the L2 larval stage in *S. frugiperda* corn and rice strain caterpillars fed on artificial diet and maize, (C) *in vitro* enzyme activity assays towards DIMBOA from midgut extracts prepared from *S. frugiperda* corn strain, rice strain and *S. littoralis* caterpillars fed on artificial diet and corn leaves, (D) specific activity measured towards DIMBOA from crude microsomal extracts prepared from *T. ni* insect cell cultures heterologously producing *S. frugiperda* corn strain UGT33F28 and *S. littoralis* UGT33F28 homologs (SlUGT33F28 a and b). Data are presented as mean ± SEM (n=3). One way ANOVA was performed and Holm-Sidak method was applied to carry out all pairwise comparisons. The differences between individual means are denoted as small letters (a-b).

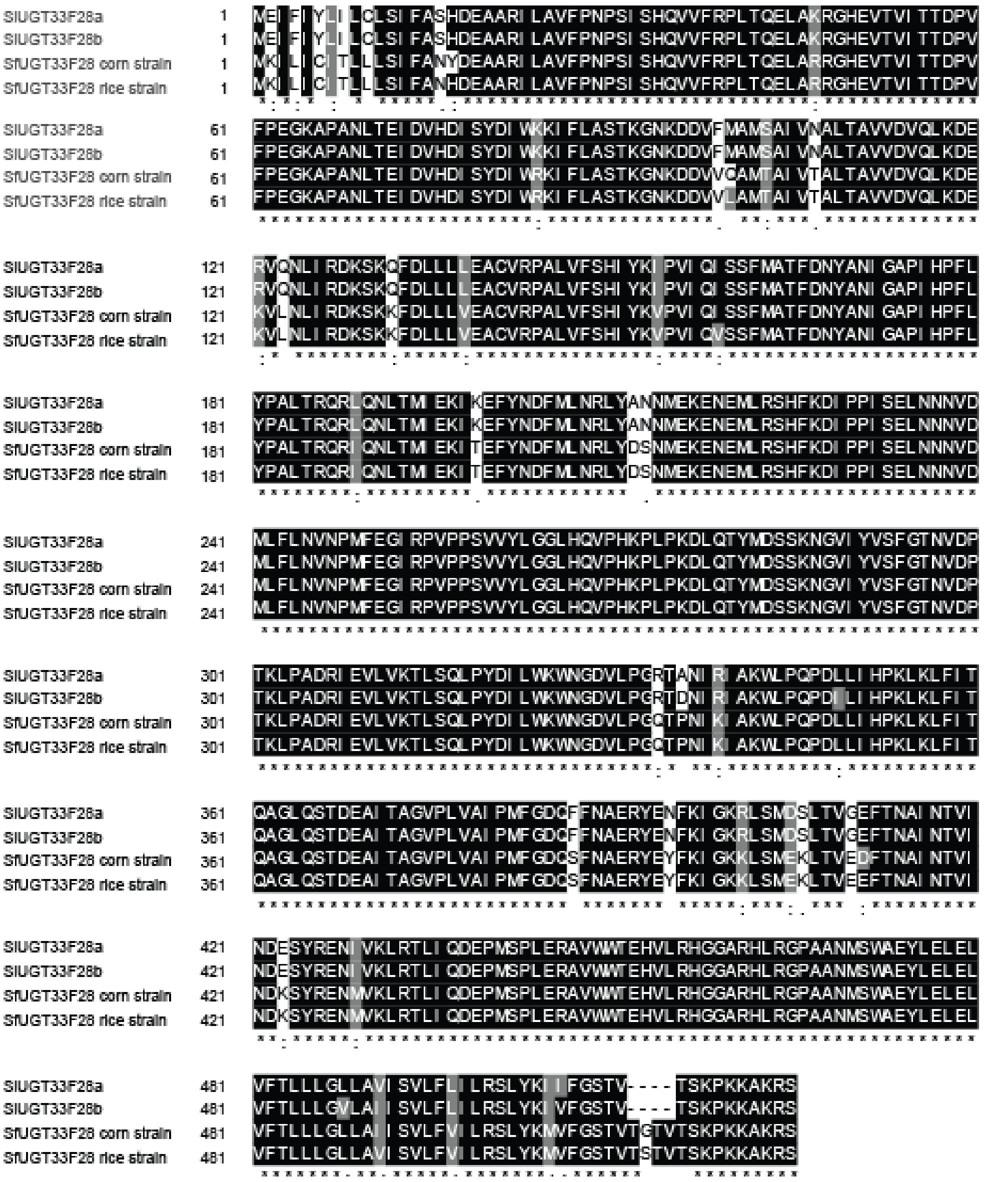


**Supplementary figure 7. Protein sequence alignment of SfUGT33F28 from *S. frugiperda* host differentiated strains with UGT33F28 homolog from *S. littoralis* using Clustal omega**.

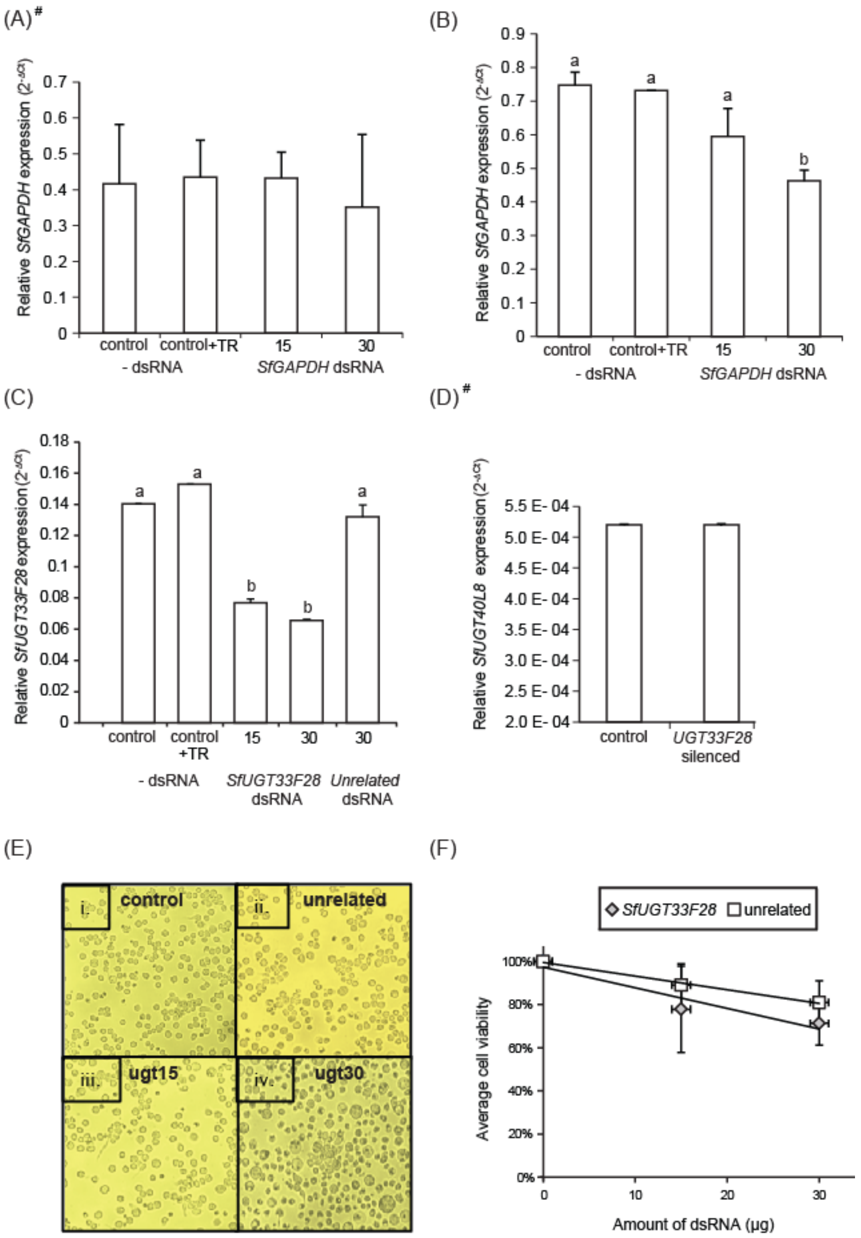


**Supplementary figure 8. Optimization of RNAi-mediated knock-down of *SfUGT33F28* in cultured Sf9 cells showing endogenous glycosylation activity**. (A-B) time course quantitative analysis of silencing on Sf9 cells treated with 15 µg and 30 µg of dsRNA against *SfGAPDH*, 24 and 48 hours post transfection (TR: controls treated with transfection reagent), (C) quantitative analysis of *SfUGT33F28* silencing on Sf9 cells transfected with 15 µg and 30 µg dsRNA specific to *UGT33F28* and an unrelated dsRNA control (TR: controls treated with transfection reagent), 48 hours post transfection, (D) quantitative *SfUGT40L8* expression analysis in control and ds*UGT33F28* transfected Sf9 cells to rule out conditional functional compensation under lack of *SfUGT33F28* gene product, (E) cell morphology of Sf9 control and ds*UGT33F28* treated cells, (F) MTT cytotoxicity assay for determination of cell viability in control and dsRNA transfected Sf9 cells. Data are presented as mean ± SEM (n= 3-5 for RNAi treatments in Sf9 cells). One way

ANOVA was performed and Tukey's test was applied for all pairwise comparisons (A, B, C). The differences between the individual means are denoted as small letters (a-b). T-tests were performed to differentiate between the means (D). #- no significant difference in expression levels among the samples.

**Supplementary table 1. *UGT* expression levels in Sf9 cells.** Illumina reads from the Sf9 transcriptome (Shu et al., 2017) were downloaded from GenBank SRA Archives SRR5892097 on November 6, 2019. CLC Genomics Workbench 12.0.4 was used for assembly of the 87 Mio paired-end (2 x 100 bp) reads, mapping, and TPM/RPKM expression calculations. For the assembly, the graph parameters were set for automatic word size, a bubble size of 150, mismatch cost of 2, and insertion and deletion cost of 3. Mappings were performed with default parameter settings except for the length fraction (0.8), the similarity fraction (0.92) and the maximum number of hits for a read (5). RPKM indicates Reads per Kilobase of transcript per Million mapped reads, a normalized index of transcript expression. n.d., not enough reads to assemble a contig for RPKM calculations. When this was the case, the number of reads was estimated by inspection of the output of NCBI blastn against the Sequence Read Archive SRR5892097. Three housekeeping genes with different expression levels are shown for comparison: *LDH* (lactate dehydrogenase), *eIF4A* (eukaryotic initiation factor 4A) and *RpL5* (ribosomal protein L5).

| **Name** | **Contig RPKM** | **Contig Reads** |
|---|---:|---:|
| *SfUGT33B24* | 4.1 | 1,181 |
| *SfUGT33B25* | 4.1 | 1,181 |
| *SfUGT33F28* | 125.5 | 115,108 |
| *SfUGT33F29* | 4.8 | 824 |
| *SfUGT33F31* | 0.8 | 226 |
| *SfUGT33F34* | 2.5 | 774 |
| *SfUGT33J12* | 3.3 | 1,075 |
| *SfUGT33S2* | 1.7 | 404 |
| *SfUGT33T9* | 1.0 | 146 |
| *SfUGT33V6* | 0.2 | 22 |
| *SfUGT33V7* | 25.5 | 23,693 |
| *SfUGT39B21* | n.d | 0 |
| *SfUGT40D16* | 2.6 | 854 |
| *SfUGT40D17* | 3.8 | 1,119 |
| *SfUGT40F17* | n.d | 11 |
| *SfUGT40F19* | 1.8 | 810 |
| *SfUGT40L8* | n.d | 13 |
| *SfUGT40M10* | n.d | 4 |
| *SfUGT40M11* | n.d | 0 |
| *SfUGT40M8* | 1.6 | 656 |
| *SfUGT40Q8* | 6.9 | 2,488 |
| *SfUGT40R11* | 8.2 | 2,946 |
| *SfUGT40R12* | 19.4 | 7,997 |

| | | |
|---|---|---|
| *SfUGT40R13* | 30.2 | 11,726 |
| *SfUGT41B7* | 6.7 | 578 |
| *SfUGT41D8* | 219.4 | 80,133 |
| *SfUGT42B13* | n.d | 6 |
| *SfUGT42C10* | n.d | 1 |
| *SfUGT44A18* | 0.3 | 17 |
| *SfUGT46A15* | 0.4 | 34 |
| *SfUGT46B5* | 0.5 | 47 |
| *SfUGT47A12* | 3.6 | 375 |
| *SfUGT48D4* | n.d | 27 |
| *SfUGT50A15* | n.d | 16 |
| *SfeIF4A* | 28.2 | 15,061 |
| *SfLDH* | 228.2 | 110,961 |
| *SfRpL5* | 1605.4 | 342,697 |

**Supplementary table 2**. **Summary of SfUGT33F28 glycosylation activities observed across a spectrum of substrates.** ANOVA was employed based on the net amounts of glucoside produced by UGT33F28 after subtracting what was produced by the corresponding amount of non-transfected control (NTC). When the glucoside formation did not differ significantly from NTC, the enzyme was considered not active (-) towards the substrate. The activity was scored on a scale of 1 to 3, 1 corresponding to the lowest and 3 corresponding to the highest activity.

| Substrate | SfUGT33F28 enzyme activity |
|---|---|
| **Benzoxazinoid-related** | |
| Indole | - |
| IAA | 3 |
| BOA | - |
| 6-OH-BOA | - |
| MBOA | - |
| DIMBOA | 3 |
| HMBOA | 3 |
| **Phenolic compounds** | |
| 4-nitrophenol | 1 |
| 1-naphthol | - |
| Salicyl aldehyde | - |
| Vanillin | 1 |
| Catechol | - |
| L-Tyrosine | - |
| L-DOPA | - |
| Dopamine | - |
| **Flavonoids** | |
| Quercetin | - |
| **Terpenoids** | |
| Menthol | - |
| Gossypol | - |
| **Coumarins** | |
| Esculetin | 1 |
| **Alkaloids** | |
| Capsaicin | - |

**Supplementary table 3. List of primers used for the amplification of full length SfUGTs described in the study.**

| *Gene* | **Forward primer (5'-3')** | **Reverse primer (5'-3')** |
|---|---|---|
| *SfUGT33V7* | AGGACACGTGCAATGTCGTTC | ACTCCTTTTAACCTTACCAGCG |
| *SfUGT40R1* | GTGCCGATAATGGCCTTAGTAT | ATTCCTTTTTTACTATTTACAATTT |
| *SfUGT33F2* | TAGAAAATGAAGATATTAATTT | ACTACGTTTGGCTTTTTTAGGTT |
| *SfUGT40D1* | ATTGAGAATATGGAAAGAATG | GTTGTTCTTCTTTTGGCTTCC |
| *SfUGT50A1* | GCAAAAATGCATAGGTGGA | CAATTTCTTCTTTGTTGCCG |
| *SfUGT40F1* | CTGTCAAAAATGGAGATAATT | ATTCTTCTTAAGTTTTGGGCTCT |
| *SfUGT41D8* | ACAATGAAGCTAAGTATCCTGT | ATTCCTTTTCTTTTTACTAGATTTCT |
| *SfUGT41B7* | CCGTTCGAAATGAAGGCT | ATTCTTCTTCTTATGCGCTAGTCT |
| *SfUGT33B2* | TTGCGTGTAATCATGTCTACCG | ATTGTCTTTTAACTTACTATATTTCA |
| *SfUGT33F3* | CGCGTTACAATGTCGGTATTAC | ATTCCGTTTCTTCTTCACACTAGTTT |
| *SfUGT42C1* | AATAAAATGAAGTTTAATAAT | CTTAGTCTTGAACTCATAATAACGA |
| *SfUGT44A1* | GCATTCAAAATGACAAAACAG | TTCGAACTTCACTCTTTTTGAGGA |
| *SfUGT40Q8* | AGCAATAAAATGAACAAATGG | ACTCCTTTTCTTCTTTTTATCTTCAC |
| *SfUGT33F3* | ATAAAAATGTTATCTTTTGTGT | ATTATTTTTAACTTTAGGAGTAGAA |
| *SfUGT40R1* | TTGTGCGTGCCAATAATGGCG | ATTCTTCTTATCCTTTTTCTTACTAT |
| *SfUGT40M1* | TGTGTCACAATGAGGTTGCCA | ATTAGTTTTCTTCTGATCATATTTTG |
| *SfUGT48D4* | GTAGCTGACATGAGGCGG | TTCGTTTTTCTTGTCTTCAAAATA |
| *SfUGT39B2* | AAAATGGACCCCCTAAAAATA | ATGTTTCTTCTGTTTTTTCGTTG |
| *SfUGT33J1* | CTCAAGATGGTTGGGTTT | ACTACTCTTCACTTTAGTCTTAATT |
| *SfUGT40L8* | TTTGTAAATATGAAGCATAAA | GTGACTCTTCTCTTTTTTATTAGTCG |
| *SfUGT33T9* | TCGATCACAATGTCTGTGC | ACTCCGTTTAATCTTAACATCACTA |
| *SfUGT42B1* | TCCATAATGAAGCCCGGC | CGTTCGCTTCGCCTTTTT |
| *SfUGT46A1* | TTGCCCACGATGCG | ATGCAACTTCTCCTTCTTATTCG |
| *SfUGT33F2* | AGCCAGACAATGAGGTATTTT | ACTACGTTTTGATTTTGATTTTTTAA |
| *SfUGT40R1* | ATTTGTAAAATGGCATTAGCAA | ATTCCTCTTCTTACTGTCTACAATTT |
| *SfUGT40M8* | AGAACAAAAATGGCGGATT | ATTCTTCTTCTCTTTTATATTCTTCT |
| *SfUGT40D1* | TCAGCCATCATGCAACG | ATTCTTCTTCTTTTTTCCTCCTGTAT |
| *SfUGT40M1* | TGTGCCGTAATGAAGTGG | CATTATTTTCTTTTTGCATATTTTCA |
| *SfUGT33B2* | CGTTCGATAATGTTCTTCCC | ATTCTTCTTAACTTTCTTCTGCTTTG |
| *SfUGT46B5* | TATCTCAAGATGCTACTCCCG | ACTCTGTTTAAGTTTCTCAGTCTTTT |
| *SfUGT47A1* | AGCACAGAGATGAGGCCG | ATTGGGTTTCACTTTGTACTTCC |
| *SfUGT40F1* | TTAATAATAATGGAAAAGTTA | ATTCTTCTTCTCTTTTATATTCTTCT |
| *SfUGT33S2* | AGTGAGAACATGTTGCTGTGC | GTCTGCCTTAAGCTTCTTTAAAGT |
| *SfUGT33V6* | GAAACCAACATGTCTCTAGTA | TGCGCGCTTAATTTTAATAATAG |

**Supplementary table 4. List of primers used for site-directed mutagenesis in the catalytic region of *SfUGT33F28*.**

| Gene | Forward primer (5'-3') | Reverse primer (5'-3') |
|---|---|---|
| *SfUGT33F28* AT-TG | ACCCTTCAATCAGCCTGCAAGTGGTTTTCCGA | TCGGAAAACCACTTGCAGGCTGATTGAAGGGT |

**Supplementary table 5. List of primers used for quantitative real time PCR (OGS2.2 gene annotation in brackets).**

| Gene | Forward primer (5'-3') | Reverse primer (5'-3') |
|---|---|---|
| *SfUGT33F28* (GSSPFG00018237001/ UGT33-13) | GTCGTTCAACGCTGAGAGATAC | CGCAGTTTTACCATGTTCTCC |
| *SfUGT33B25* (GSSPFG00024582001/ UGT33-19) | CAACGAATGAAGAGGAATTCAAG | AGGCTGGTCGTACATCACG |
| *SfUGT42C10* (GSSPFG00035369001/ UGT42-01-hap) | CCGCTGCTATCGAAGAAAAC | TCATGCCATACTTTGGATCG |
| *SfUGT40L8* (GSSPFG00008466001/ UGT40Lb) | AGCAAACATCGCTGGTTACC | TAAATGACGCCGTGTTTCGC |
| *SfUGT40R13* (GSSPFG00014601001/ GSSPFG00005183001/ UGT40-05) | TCGCTAGAGGCTTTGCTTTG | TCGGTCGTGGTAGATGTAGG |
| *SfUGT46A15* (GSSPFG00032149001/ GSSPFG00021909001/ UGT46-01) | TCGAAGGATTGCAGAGTGTG | TGCTGTGTCCAAAGGATCTG |
| *SfGAPDH* (GSSPFG00028139001/ GSSPFG00007681001) | TGGATAACTTTGGCGAGAGG | TGTCAACCTTGACGCTTACG |
| *SfRPL10* (GSSPFG00012205001) | ATTGGACAGCCCATCATGTC | AAGCCCCATTTCTTGGAGAC |

**Supplementary table 6. List of primers used for the preparation of RNAi constructs.**

| Gene | Forward primer (5'-3') | Reverse primer (5'-3') |
|---|---|---|
| *SfUGT33F28* RNAi | TAATACGACTCACTATAGGGCCAAGTACCTCATAAACCAT | CCCTATAGTGAGTCGTATTAGTCCAGGCAGAACATCA |
| *SfGAPDH* RNAi | TAATACGACTCACTATAGGGGAAGGGCATTCTTGACTA | CCCTATAGTGAGTCGTATTATTGGTCTGGATGTACTTG |


Shu, B., Zhang, J., Sethuraman, V., Cui, G., Yi, X., and Zhong, G. (2017). Transcriptome analysis of Spodoptera frugiperda Sf9 cells reveals putative apoptosis-related genes and a preliminary apoptosis mechanism induced by azadirachtin. *Scientific reports* 7**,** 1-13.
